# Lentiviral haematopoietic stem-cell gene therapy for early-onset metachromatic leukodystrophy: long-term results from a non-randomised, open-label, phase 1/2 trial and expanded access

**DOI:** 10.1016/S0140-6736(21)02017-1

**Published:** 2022-01-22

**Authors:** Francesca Fumagalli, Valeria Calbi, Maria Grazia Natali Sora, Maria Sessa, Cristina Baldoli, Paola Maria V Rancoita, Francesca Ciotti, Marina Sarzana, Maddalena Fraschini, Alberto Andrea Zambon, Serena Acquati, Daniela Redaelli, Vanessa Attanasio, Simona Miglietta, Fabiola De Mattia, Federica Barzaghi, Francesca Ferrua, Maddalena Migliavacca, Francesca Tucci, Vera Gallo, Ubaldo Del Carro, Sabrina Canale, Ivana Spiga, Laura Lorioli, Salvatore Recupero, Elena Sophia Fratini, Francesco Morena, Paolo Silvani, Maria Rosa Calvi, Marcella Facchini, Sara Locatelli, Ambra Corti, Stefano Zancan, Gigliola Antonioli, Giada Farinelli, Michela Gabaldo, Jesus Garcia-Segovia, Laetitia C Schwab, Gerald F Downey, Massimo Filippi, Maria Pia Cicalese, Sabata Martino, Clelia Di Serio, Fabio Ciceri, Maria Ester Bernardo, Luigi Naldini, Alessandra Biffi, Alessandro Aiuti

**Affiliations:** a San Raffaele Telethon Institute for Gene Therapy (SR-Tiget), IRCCS San Raffaele Scientific Institute, Milan, Italy; b Pediatric Immunohematology Unit and BMT Program, IRCCS San Raffaele Scientific Institute, Milan, Italy; c Units of Neurology and Neurophysiology, IRCCS San Raffaele Scientific Institute, Milan, Italy; d Unit of Neurorehabilitation, IRCCS San Raffaele Scientific Institute, Milan, Italy; e Neuroradiology Unit, IRCCS San Raffaele Scientific Institute, Milan, Italy; f Clinical Molecular Biology Laboratory, IRCCS San Raffaele Scientific Institute, Milan, Italy; g Department of Anesthesia and Critical Care, IRCCS San Raffaele Scientific Institute, Milan, Italy; h Unit of Hematology and Bone Marrow Transplantation, IRCCS San Raffaele Scientific Institute, Milan, Italy; i Neuroimaging Research Unit, Division of Neuroscience, IRCCS San Raffaele Scientific Institute, Milan, Italy; j University Centre of Statistics in the Biomedical Sciences (CUSSB), Milan, Italy; k Vita-Salute San Raffaele University, Milan, Italy; l Biomedical Faculty, Università della Svizzera Italiana, Lugano, Switzerland; m Department of Chemistry, Biology and Biotechnologies, University of Perugia, Perugia, Italy; n Orchard Therapeutics (Europe), London, UK; o Department of Specialistic Neurological Rehabilitation, IRCCS Multimedica, Sesto San Giovanni, Italy; p Department of Neurology, ASST Papa Giovanni XXIII Bergamo, Italy; q Division of Pediatric Hematology, Oncology and Stem Cell Transplant, Padua University and Padua University Hospital, Padua, Italy; r Gene Therapy Program, Dana Farber/Boston Children's Cancer and Blood Disorders Center, Boston, MA, USA

## Abstract

**Background:**

Effective treatment for metachromatic leukodystrophy (MLD) remains a substantial unmet medical need. In this study we investigated the safety and efficacy of atidarsagene autotemcel (arsa-cel) in patients with MLD.

**Methods:**

This study is an integrated analysis of results from a prospective, non-randomised, phase 1/2 clinical study and expanded-access frameworks. 29 paediatric patients with pre-symptomatic or early-symptomatic early-onset MLD with biochemical and molecular confirmation of diagnosis were treated with arsa-cel, a gene therapy containing an autologous haematopoietic stem and progenitor cell (HSPC) population transduced ex vivo with a lentiviral vector encoding human arylsulfatase A (ARSA) cDNA, and compared with an untreated natural history (NHx) cohort of 31 patients with early-onset MLD, matched by age and disease subtype. Patients were treated and followed up at Ospedale San Raffaele, Milan, Italy. The coprimary efficacy endpoints were an improvement of more than 10% in total gross motor function measure score at 2 years after treatment in treated patients compared with controls, and change from baseline of total peripheral blood mononuclear cell (PBMC) ARSA activity at 2 years after treatment compared with values before treatment. This phase 1/2 study is registered with ClinicalTrials.gov, NCT01560182.

**Findings:**

At the time of analyses, 26 patients treated with arsa-cel were alive with median follow-up of 3·16 years (range 0·64–7·51). Two patients died due to disease progression and one due to a sudden event deemed unlikely to be related to treatment. After busulfan conditioning, all arsa-cel treated patients showed sustained multilineage engraftment of genetically modified HSPCs. ARSA activity in PBMCs was significantly increased above baseline 2 years after treatment by a mean 18·7-fold (95% CI 8·3–42·2; p<0·0001) in patients with the late-infantile variant and 5·7-fold (2·6–12·4; p<0·0001) in patients with the early-juvenile variant. Mean differences in total scores for gross motor function measure between treated patients and age-matched and disease subtype-matched NHx patients 2 years after treatment were significant for both patients with late-infantile MLD (66% [95% CI 48·9–82·3]) and early-juvenile MLD (42% [12·3–71·8]). Most treated patients progressively acquired motor skills within the predicted range of healthy children or had stabilised motor performance (maintaining the ability to walk). Further, most displayed normal cognitive development and prevention or delay of central and peripheral demyelination and brain atrophy throughout follow-up; treatment benefits were particularly apparent in patients treated before symptom onset. The infusion was well tolerated and there was no evidence of abnormal clonal proliferation or replication-competent lentivirus. All patients had at least one grade 3 or higher adverse event; most were related to conditioning or to background disease. The only adverse event related to arsa-cel was the transient development of anti-ARSA antibodies in four patients, which did not affect clinical outcomes.

**Interpretation:**

Treatment with arsa-cel resulted in sustained, clinically relevant benefits in children with early-onset MLD by preserving cognitive function and motor development in most patients, and slowing demyelination and brain atrophy.

**Funding:**

Orchard Therapeutics, Fondazione Telethon, and GlaxoSmithKline.

**Research in context**

**Evidence before this study**

We searched PubMed from database inception to Feb 8, 2021, for relevant studies of treatments for metachromatic leukodystrophy (MLD), including acronyms, synonyms, and closely related words for the terms “metachromatic leukodystrophy (MLD)”, “therapy”, “enzyme replacement therapy (ERT)”, and “gene therapy (GT)”. We did not restrict our search by study design or language. We identified further studies by searching relevant websites and the reference lists of all included articles identified by our search. Evidence of allogeneic haematopoietic stem-cell transplantation being beneficial in patients with late-infantile MLD appears to be very scarce. Children treated when pre-symptomatic might show delayed disease onset and attenuated progression compared with untreated patients, but still have severe peripheral neuropathy and severe impairment of gross motor function post-treatment. Furthermore, allogeneic haematopoietic stem-cell transplantation did not slow down disease progression in patients with early-onset MLD with overt neurological symptoms at the time of transplantation. Outcomes in juvenile and adult MLD patients are more favourable, with long-term stabilisation of neurological symptoms, but are associated with conditioning-related complications and graft-versus-host disease. Intracerebral gene therapy with an adeno-associated viral vector has been tested in the context of a clinical trial but did not reverse the fatal outcome of the disease (NCT01801709). Preliminary results on the safety and tolerability of intrathecal enzyme replacement therapy have been recently published but the efficacy of this treatment in halting disease progression has yet to be shown. Contrarily, haematopoietic stem and progenitor cell gene therapy (HSPC-GT) consisting of an autologous transplant of haematopoietic stem and progenitor cells (HSPCs) transduced ex vivo with a lentiviral vector encoding the *ARSA* gene, resulted in prevention and correction of CNS and peripheral nervous system in the mouse model. A non-randomised, single arm, phase 1/2 clinical study on HSPC-GT combined with a myeloablative conditioning regimen was therefore initiated in patients with early-onset MLD in 2010 (NCT01560182). Preliminary results for the first three patients with the late-infantile variant who took part in the trial were reported in 2013. The safety and efficacy profile of HSPC-GT in early-onset MLD was expanded in a paper published in 2016, describing the first nine patients treated, indicating a favourable outcome in late-infantile patients treated in a pre-symptomatic phase, with a maximum follow-up of 54 months.

**Added value of this study**

This study provides key and novel information on the safety profile of HSPC-GT and the long-term efficacy in a large cohort of 29 patients with early-onset MLD treated in the context of the phase 1/2 study and expanded access frameworks and followed up for up to 7·5 years. All treated patients showed high and sustained engraftment of corrected cells that was associated with increased, sustained ARSA activity in peripheral blood and cerebrospinal fluid. HSPC-GT provided benefit in pre-symptomatic late-infantile and pre-symptomatic and early-symptomatic early-juvenile patients treated before entering the rapid phase of disease progression, preserving cognitive function, delaying time to severe motor disability, and slowing down brain demyelination and atrophy.

**Implications of all the available evidence**

This study shows the long-term benefits of administering a lentiviral-based gene therapy to patients with MLD, for whom there are currently no effective treatment options. On the basis of the interim analyses presented in this work, HSPC-GT has recently been granted full marketing authorisation in Europe by the European Commission and in the UK by the Medicines and Healthcare products Regulatory Agency (Libmeldy) and is under investigation in the USA. We believe that the findings from this study represent an important advance in the treatment of the most severe variants of MLD, and its efficacy is currently being further studied in the late-juvenile variant of MLD (NCT04283227).

## Introduction

Metachromatic leukodystrophy (MLD) is a rare inherited lysosomal storage disease caused by deficiency of arylsulfatase A (ARSA), due to mutations in the *ARSA* gene.^1^ Reduced ARSA activity results in accumulation of sulfatides in the CNS and peripheral nervous system, leading to progressive dysmyelination, neuroinflammation, and neurodegeneration.^1^, ^2^, ^3^ These events result in progressive motor and cognitive deterioration, with loss of motor and neurocognitive functions, and ultimately death.^1^, ^2^, ^4^ Sulfatides also accumulate in visceral organs, such as the gallbladder and kidneys.^5^, ^6^

The clinical spectrum of MLD is broad and heterogeneous. Three clinical forms are commonly described on the basis of age at first symptom onset: late-infantile (≤30 months), juvenile (subdivided into early juvenile [30 months–6 years] and late juvenile [7–16 years]), and adult MLD (≥17 years), with earlier age at onset or presence of motor symptoms at onset associated with a more severe and rapid disease course.^1^, ^2^, ^3^, ^7^, ^8^ Regardless of the clinical variant, the underlying disease pathophysiology is similar for all phenotypic forms of MLD.^1^, ^4^, ^9^

There is a high unmet need for effective therapies for MLD, particularly in the early-onset variants (age <7 years at onset) for which disease management is mainly focused on palliative care. Allogeneic haematopoietic stem-cell transplantation has shown poor efficacy, particularly in early-onset MLD, and in halting progression of peripheral demyelination.^10^, ^11^, ^12^, ^13^ For other experimental therapies, including enzyme replacement therapy and CNS-administered adeno-associated virus gene therapy, preliminary clinical data showed no or little efficacy.^14^, ^15^

Atidarsagene autotemcel (arsa-cel) is a gene therapy medicinal product based on autologous haematopoietic stem and progenitor cells (HSPCs) transduced ex vivo with a lentiviral vector encoding the *ARSA* cDNA driving supranormal ARSA expression in HSPCs and their progeny, which has previously shown preliminary evidence of safety and efficacy in nine patients with early-onset MLD.^16^, ^17^ In this Article, we present results from 29 patients with early-onset MLD (16 with late-infantile MLD, 13 with early-juvenile MLD) treated with arsa-cel, with maximum follow-up of 7·5 years.

## Methods

### Study design

We undertook an integrated analysis of results from a prospective, non-randomised, phase 1/2 clinical study and expanded-access frameworks (appendix p 4). Treated patients were compared with a historical cohort of 31 patients with early-onset MLD from a non-interventional natural history (NHx) study (patients enrolled from 2004, data collected with retrospective and prospective assessments from 2000 to 2017) to assess the safety and efficacy of arsa-cel.^18^ All studies had comparable endpoints and schedules of assessments.^9^, ^16^, ^17^ Important protocol deviations are detailed in the appendix (p 4). Patients were treated and followed up at Ospedale San Raffaele, Milan, Italy.

All studies were undertaken in accordance with the principles of Good Clinical Practice and the Declaration of Helsinki and with approval of the Ospedale San Raffaele Ethics Committee and Agenzia Italiana del Farmaco, where applicable. Informed consent was obtained from the patients’ parents or guardians.

### Patients

Eligible patients had a molecular and biochemical diagnosis of MLD of either pre-symptomatic late-infantile or pre-symptomatic or early-symptomatic early-juvenile variants, as detailed in the appendix (p 10). Late-infantile MLD was diagnosed when predicted age at symptom onset was 30 months or less of chronological age, based on the index sibling. Early-juvenile MLD was diagnosed when predicted age at onset based on the index sibling or the patient's own age at disease onset was between 30 months and 6 years. Early-symptomatic status was initially defined as presence of symptoms for less than 6 months. The definition was subsequently amended in January, 2014, to allow the treatment of patients with an intelligence quotient (IQ) of 70 or greater and the ability to walk ten or more steps independently, and to prevent enrolment of severely impaired patients or those entering a rapid phase of disease progression for whom benefit from treatment was not expected.

### Procedures

Patients were treated and monitored according to the schedule described in the appendix (p 17) and as previously reported.^16^, ^17^ HSPCs harvested from bone marrow or mobilised peripheral blood (MPB) were transduced with clinical-grade lentiviral vector encoding human *ARSA* cDNA under the control of the human phosphoglycerate kinase gene promoter.^16^ Before intravenous infusion of arsa-cel, patients received busulfan conditioning. Busulfan was initially administered at a target cumulative area under the curve (AUC) of 67·2 mg × h/L (submyeloablative; actual range 63·4–84·3 mg × h/L; n=13) and then increased to a target AUC of 85·0 mg × h/L (myeloablative; actual range 78·0–88·3 mg × h/L; n=16) with the goal of reducing the variability of transduced cell engraftment observed in the first nine patients treated (for further details, see appendix p 8). The actual median busulfan cumulative AUC overall was 79·9 mg × h/L (range 63·4–88·3; n=29).

### Outcome measures

The coprimary efficacy endpoints were an improvement of more than 10% (considered a clinically relevant change in response to treatment in different settings)^19^ in the total score of the gross motor function measure (GMFM)^19^ at 2 years after treatment in treated patients compared with controls matched by age and disease subtype, and change from baseline of total peripheral blood mononuclear cell (PBMC) ARSA activity at 2 years after treatment compared with values before treatment. The gross motor function measure instrument used in this study (GMFM-88)^19^ consists of 88 items organised into five domains: lying and rolling; sitting; crawling and kneeling; standing; and walking, running, and jumping. Each of the 88 questions is scored from 0 to 3 (maximum number of points=264) and a composite percentage score (0–100%) is calculated from the total score, with 0% corresponding to loss of all voluntary movement. The GMFM score is also related to age; by the age of 60 months most healthy children will achieve their maximum score, approximating 100%.

Other endpoints included the percentage of lentiviral vector-positive bone marrow-derived clonogenic progenitor cells, ARSA activity in cerebrospinal fluid on soluble enzyme from ion-exchange chromatography,^20^ gross motor function classification for MLD (GMFC-MLD) at different ages, and brain MRI total score and nerve conduction velocity index at 2 and 3 years after treatment in treated patients compared with controls matched by age and disease subtype. A supplementary set of exploratory analyses were also specified for the integrated dataset (severe motor impairment-free survival and cognitive age-equivalent profiles) and are detailed in the appendix (p 7). Safety endpoints included conditioning regimen-related safety, short-term and long-term safety of lentiviral vector-transduced cell infusion, and monitoring of adverse events and laboratory values in treated patients. A detailed list of study endpoints is available in the appendix (p 12).

### Statistical analysis

Total scores for GMFM were compared with NHx study participants matched by age and disease subtype using an analysis of covariance model adjusted for treatment and age at assessment of gross motor function measure. ARSA activity in PBMCs was analysed using a mixed-model repeated measures model. The safety population, comprising all arsa-cel treated patients, was analysed descriptively. All statistical analyses were two-sided and undertaken at the 5% significance level. Further details of the statistical methods for the integrated efficacy and safety analyses are provided in the appendix (p 7).

### Role of the funding source

The study was originally designed by San Raffaele Telethon Institute of Gene Therapy investigators and sponsored by Ospedale San Raffaele and Fondazione Telethon until 2014, when financial sponsorship was transferred from Telethon to GlaxoSmithKline. Full sponsorship was then transferred to GlaxoSmithKline in 2016, and subsequently to Orchard Therapeutics in 2018. Data were collected by trial investigators with analysis conducted through collaboration between the sponsor and the principal investigators.

## Results

Integrated analyses were done on 29 patients treated with arsa-cel (primary study, n=20 [69%]; expanded-access frameworks, n=9 [31%]) from May, 2010, to October, 2017, according to the timeline described in the appendix (p 4). 16 (55%) patients had late-infantile MLD (one was pre-symptomatic at enrolment and became symptomatic by time of treatment) and 13 (45%) had early-juvenile MLD (eight [28%] were early-symptomatic at treatment). 26 of 29 (90%) patients were alive at the time of data cutoff. Median follow-up was 3·16 years (range 0·64–7·51). The table shows patients’ baseline characteristics, and patients’ disposition and drug product characteristics are described in appendix pp 18 and 13, respectively. All patients received bone marrow-derived CD34^+^ cells apart from two patients who were infused additionally with MPB-derived CD34^+^ cells and one patient with MPB-derived CD34^+^ cells only; the drug product characteristics and transduction efficiency obtained with MPB as a cell source was within the range observed for that obtained with bone marrow (appendix p 13).

**Table:** Baseline characteristics in the ITT set and natural history cohort

		**ITT set**		**Natural history cohort**	
		Late infantile (n=16)	Early juvenile (n=13)	Late infantile (n=19)	Early juvenile (n=12)
Pre-symptomatic		15 (94%)	5 (38%)	0	0
Early-symptomatic*		1† (6%)	8 (62%)‡	19 (100%)	12 (100%)
Mean age at GT (ITT set) or initial assessment (natural history cohort), months (SD)		12·81 (4·3)	65·86 (33·4)	20·64 (4·7)	51·98 (19·2)
Median follow-up, years (range)		3·04 (0·99–7·51)	3·49 (0·64–6·55)	4·54 (1·80–14·19)	6·79 (2·51–16·10)
Female sex		6 (38%)	7 (54%)	11 (58%)	7 (58%)
Race					
	Asian (South-East Asian heritage)	1 (6%)	0	0	0
	White (Arabic/North African heritage)	4 (25%)§	0	3 (16%)§	0
	White (White/Caucasian European)	11 (69%)§	13 (100%)	16 (84%)§	12 (100%)
MLD variants in matched populations					
	Matched analysis set (n)	16	13	17	12
	Matched sibling analysis set (n)	8	4	7	4

Data are number (%), mean (SD), or median (range), unless otherwise indicated. GT=gene therapy. ITT=intention-to-treat. MLD=metachromatic leukodystrophy.

* Symptomatic at time of treatment (for atidarsagene autotemcel [arsa-cel]) or at time of enrolment (for natural history).

† One patient with late-infantile MLD was pre-symptomatic at time of enrolment but showed disease progression between enrolment and treatment.

‡ Two patients with early-symptomatic early-juvenile MLD were enrolled according to the original inclusion criteria, one of them showing disease progression between enrolment and treatment.

§ Two patients with late-infantile MLD in the ITT set and one patient with late-infantile MLD in the natural history cohort were incorrectly coded as White-White/Caucasian European in the clinical database. After database lock, it was confirmed that these patients are White-Arabic/North African heritage, and the table reflects the correct classification.

Three deaths occurred during follow-up. Two were due to rapid disease progression in patients with early-symptomatic early-juvenile MLD (at 8 and 15 months after treatment), and were considered unrelated to arsa-cel. One was due to ischaemic stroke following an infectious event 13·6 months after treatment in a patient with pre-symptomatic early-juvenile MLD. The patient had a normal neurological examination and neuroimaging, and normal motor and cognitive development at 1 year follow-up. The event occurred away from the treatment centre. A clinical summary was provided; however, there was no available post-mortem examination. Integration site analyses did not show signs of clonal expansion or clonal dominance (appendix pp 8–9). On the basis of the available, albeit limited, data, the working cause of death was ischaemic stroke that had been deemed by the study investigators not related to the treatment.

All patients achieved haematological engraftment. Median duration of neutropenia (absolute neutrophil count <500 cells per μL) was 28 days (range 13–39). The most frequently reported grade 3 or higher adverse events were febrile neutropenia (n=23, 79%), gait disturbance (n=15, 52%), and stomatitis (n=12, 41%). Most adverse events were associated with busulfan conditioning or MLD disease progression (appendix pp 15–16). Three (10%) patients had events of veno-occlusive disease and two (7%) had events of thrombotic microangiopathy associated with conditioning; one (3%) patient required unmanipulated autologous back-up bone marrow infusion to support haematological recovery.^21^ The patient remains in good clinical condition and with stable gene marking within the range of other treated patients (after an expected transient drop following back-up infusion) at latest follow-up (2·72 years). Two (7%) patients had metabolic acidosis (one [3%] life-threatening), which resolved after specific treatment, and two (7%) had gallbladder polyps requiring cholecystectomy, all considered related to the underlying disease.^5^, ^6^

Five treatment-related events of anti-ARSA antibodies were reported in four (14%) patients, which resolved spontaneously or after B-cell depleting (rituximab) therapy, with no obvious effect on clinical outcome or safety profile (appendix p 9). The anti-ARSA antibody titres were usually low, and in three (10%) patients they were associated with presence of other autoantibodies or clinical manifestations of veno-occlusive disease or thrombotic microangiopathy.

To date, there has been no evidence of malignant clonal expansion, replication-competent lentivirus, or adverse events indicative of oncogenic transformation with arsa-cel. Insertion site analyses indicated an overall polyclonal reconstitution pattern, without evidence of clonal dominance or proliferation.^22^

**Figure 1:**
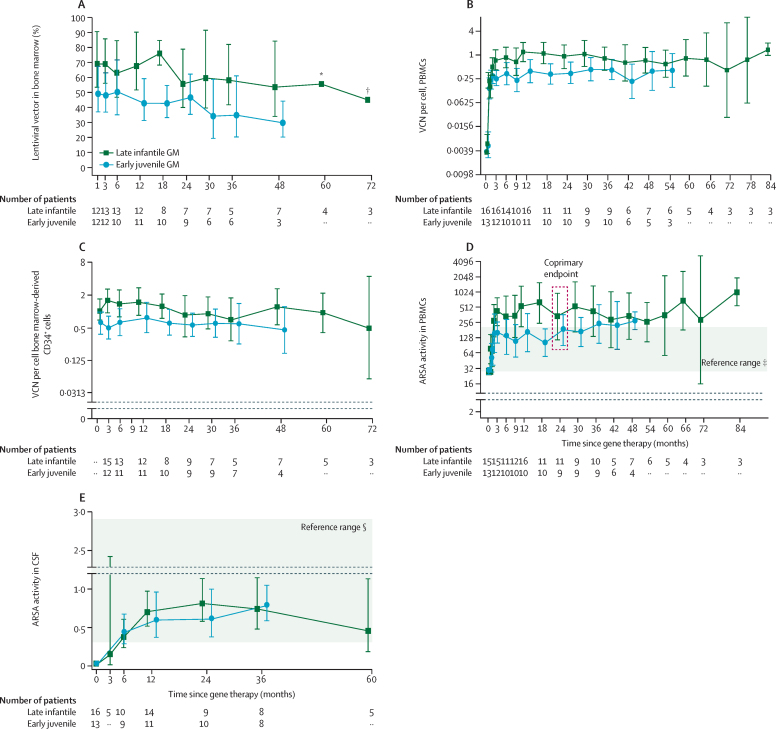
High-level engraftment of gene-corrected HSPCs (A) Assessed over time by disease subtype by percentage of lentiviral vector-transduced cells in bone marrow clonogenic progenitors. (B) Mean VCN in PBMCs. LLQ is 0·0037 VCN per cell. Zero values are plotted as 0·001. (C) Mean VCN in bone marrow-derived CD34^+^ cells. LLQ is 0·0037 vector copy number/cell. Zero values are plotted as 0·001. (D) Mean ARSA activity in PBMCs. LLQ is 25·79 nmol/mg/h. ARSA activity measured in PBMC in the intention-to-treat set after treatment at years 2 (coprimary endpoint) and 3 was compared with pre-treatment values using a mixed-model repeated measures model. (E) Mean ARSA activity in cerebrospinal fluid (CSF). LLQ is 0·0032 nmol/mg/h. Geometric means and 95% CIs are presented where there are at least three patients with non-missing data. ARSA=arylsulfatase A. GM=geometric mean. LLQ=lower limit of quantification. PBMCs=peripheral blood mononuclear cells. VCN=vector copy number. *95% CI for 60-month timepoint: 24·35–128·73. †95% CI for 72-month timepoint: 6·69–303·48. ‡From adult reference donors. §From paediatric reference donors.^20^ In all panels, values less than the LLQ were imputed as the LLQ as this represents a conservative approach to treatment evaluation in those cases.

The mean percentage of lentiviral vector-positive bone marrow clonogenic progenitors 1 year after treatment was 55% (range 20–100%), exceeding the protocol-defined target of 4%, and was stably persistent throughout follow-up (figure 1A). All patients had high and stable vector copy number values in PBMCs (figure 1B) and bone marrow-derived CD34^+^ cells (figure 1C), and in myeloid-lineage (appendix p 19) and lymphoid-lineage (appendix p 19) subpopulations. The three patients who received a drug product derived from MPB alone or in combination with bone marrow showed similar engraftment of gene-corrected cells (appendix pp 20–21). All patients showed reconstituted ARSA activity in PBMCs within or above normal range from 3 months after treatment (figure 1D), including CD14^+^ and CD15^+^ myeloid cells (appendix p 22). ARSA activity in PBMCs was significantly increased above baseline 2 years after treatment by a mean 18·7-fold (95% CI 8·3–42·2; p<0·0001) in patients with late-infantile disease and 5·7-fold (2·6–12·4; p<0·0001) in patients with early-juvenile disease, and sustained throughout follow-up (figure 1D). We noted no relevant difference in the level of ARSA activity in PBMCs between patients with late-infantile and early-juvenile MLD.

Mean ARSA activity in cerebrospinal fluid, undetectable at baseline, was above the level of quantification by the first post-baseline measurement at 3 months after treatment, reached normal levels by 6–12 months, and remained within normal range throughout available follow-up (year 3 for early-juvenile patients; year 5 for late-infantile patients; figure 1E).

A subanalysis of pharmacodynamic outcomes by conditioning regimen (submyeloablative or myeloablative) showed similar percentages of lentiviral vector-transduced HSPCs (or their progeny) measured over time in bone marrow (appendix p 23), with no differences observed in the level of transduced cell engraftment, as measured by vector copy number in bone marrow and PBMCs (appendix p 24), and ARSA activity in total PBMCs and bone marrow-derived mononuclear cells (appendix p 25). The level of busulfan exposure did not have a notable effect on ARSA activity in cerebrospinal fluid, with persistent levels within normal range in both regimens. However, the interpretation of these results could be confounded by the disproportionate representation of patients with late-infantile MLD in the submyeloablative subgroup and the small difference in exposure between regimens.

**Figure 2:**
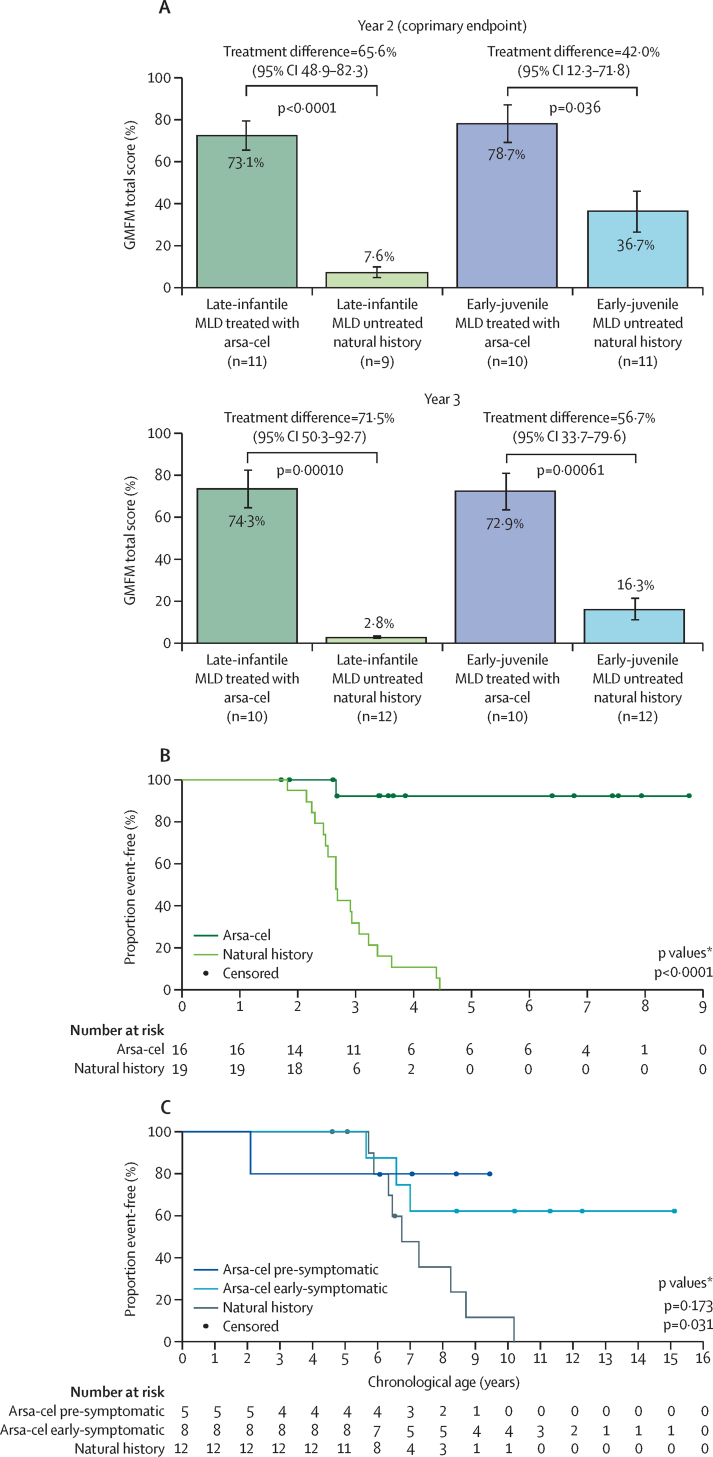
GMFM scores and age at severe motor impairment or death (A) GMFM scores for patients with late-infantile and early-juvenile MLD compared with age-matched and disease subtype-matched untreated natural history controls at 2 and 3 years after treatment. Adjusted least-squares means, treatment difference (atidarsagene autotemcel [arsa-cel] minus natural history), and the associated 95% CI and p value were reported overall and by disease subtype (late infantile, early juvenile) for the null hypothesis of 10% or less difference in total GMFM scores between treated and natural history patients at years 2 and 3. (B) Kaplan-Meier plot showing age at severe motor impairment or death in patients with late-infantile MLD versus untreated natural history late-infantile MLD controls. (C) Kaplan-Meier plot showing age at severe motor impairment or death in patients with pre-symptomatic and early-symptomatic early-juvenile MLD versus untreated natural history early-juvenile MLD controls. Severe motor impairment-free survival is defined as the interval from birth to the earlier loss of locomotion and sitting without support (GMFC level 5 or 6) or death from any cause; otherwise severe motor impairment-free survival is censored at the last GMFC assessment date. Symptomatic status refers to arsa-cel treated patients at the time of treatment. GMFM=gross motor function measure. MLD=metachromatic leukodystrophy. *p values calculated using an unstratified log-rank test.

Mean differences in total scores for gross motor function measure between treated patients and age-matched and disease subtype-matched NHx patients 2 years after treatment far exceeded the predefined minimum threshold for clinically meaningful efficacy (10%) and were significant for both patients with late-infantile MLD (66% [95% CI 48·9–82·3]) and early-juvenile MLD (42% [12·3–71·8]; figure 2A). The difference was larger at 3 years for patients with both late-infantile and early-juvenile MLD overall (figure 2A), and patients with early-juvenile MLD stratified by symptomatic status at time of treatment (appendix p 26). Furthermore, 25 of 29 treated patients displayed gross motor development similar to or slightly below normally developing children, or stabilisation of motor performance or delay in the rate of motor decline compared with NHx patients (appendix pp 27–30).

Severe motor impairment-free survival (defined as surviving and maintaining gross motor function classification level 4 or less, able to sit, or crawl or roll, or better; appendix p 17) was maintained in most treated patients regardless of disease subtype and symptomatic status at time of treatment. At 4·5 years of age, an estimated 92% (95% CI 57–99%) of treated late-infantile patients remained event-free versus 0% for NHx patients (figure 2B). At 8 years of age, an estimated 80% (95% CI 20–97%) of pre-symptomatic early-juvenile and 63% (23–86%) of early-symptomatic early-juvenile treated patients remained event-free versus 36% (9–65%) for NHx (figure 2C). Moreover, 88% of treated late-infantile patients maintained gross motor function classification level 2 or less (ability to walk) throughout available follow-up (appendix p 29).

**Figure 3:**
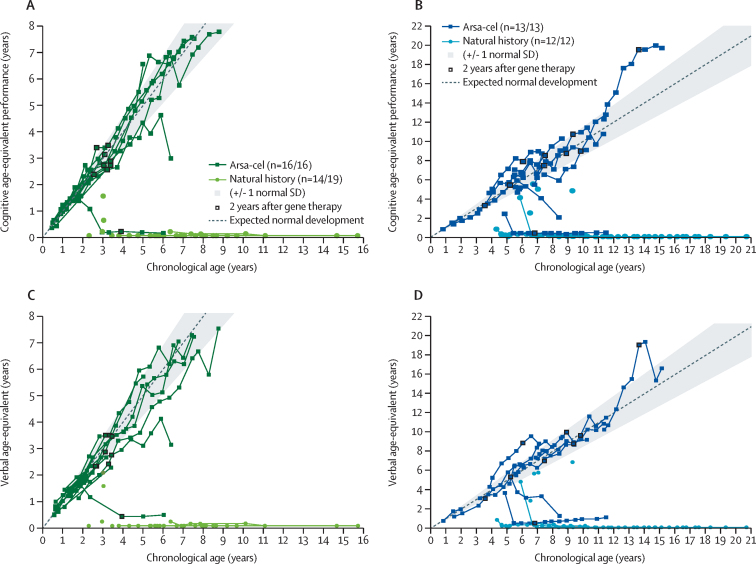
Cognitive performance and verbal age-equivalent profiles (A) Age-equivalent cognitive performance in late-infantile patients. (B) Age-equivalent cognitive performance in early-juvenile patients. (C) Verbal-age equivalent in late-infantile patients. (D) Verbal-age equivalent in early-juvenile patients. Age-equivalent corresponds to the chronological age at which, on average, typically developing children reach a given raw score. Cognitive age-equivalent for late-infantile (A) and early-juvenile (B) at each visit has been derived as follows. For Wechsler Preschool & Primary Scale of Intelligence (WPPSI) and Wechsler Intelligence Scale for Children (WISC): (development quotient performance x chronological age)/100 for which development quotient is derived by dividing the age-equivalent by the chronological age and then multiplying by 100. For Bayley III: cognitive raw scores have been compared with the tabulated values in the Bayley III manual to calculate cognitive age-equivalent. For Bayley II and in cases for which a neuropsychological assessment has been done but a questionnaire could not be completed because of severe clinical condition, cognitive age-equivalent is based on mental development age as reported on the case report form (CRF). Verbal age-equivalent for late-infantile (C) and early-juvenile (D) at each visit has been derived as follows. For WPPSI and WISC: (development quotient language x chronological age)/100. For Bayley III: expressive communication and receptive communication raw scores have each been compared with the tabulated values in the Bayley III manual and based on the average of the mental development ages for the two scores. For Bayley II and in cases for which a neuropsychological assessment has been done but a questionnaire could not be completed because of severe clinical condition, verbal age-equivalent is based on mental development age as reported on the CRF. Arsa-cel=atidarsagene autotemcel. MLD=metachromatic leukodystrophy.

Performance and verbal IQs remained within normal range throughout follow-up in most treated patients. Specifically, age-equivalent scores showed normal acquisition of cognitive skills in 20 of 25 (80%) assessed patients at similar chronological ages at which NHx patients showed severe cognitive impairment (figure 3).

**Figure 4:**
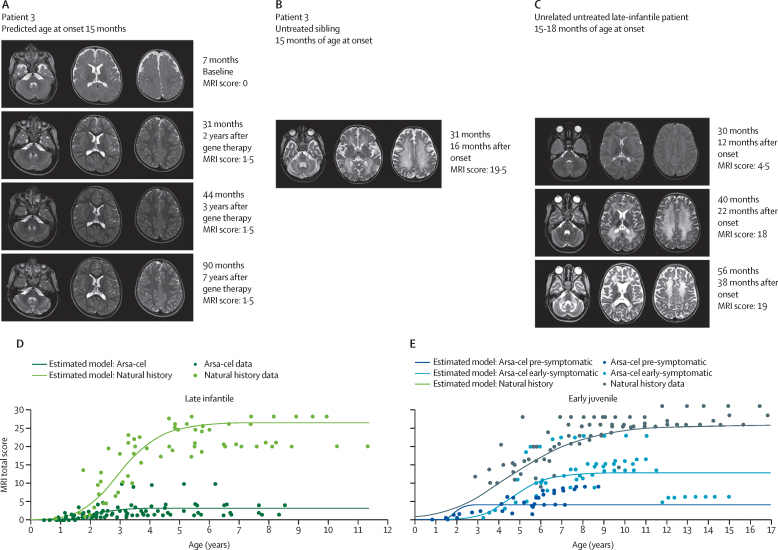
Longitudinal evaluation of brain MRI of patients treated with arsa-cel *vs* natural history (A) Axial T2-weighted MRI obtained from a patient with late-infantile MLD (patient 3) over time showing, at baseline, a physiological T2 signal corresponding to myelin maturation, typical of the first year of life, followed by appearance of subtle posterior periventricular T2 hyperintensities that stabilised over time, with normal maturation of the remaining brain regions. (B) The comparison with the corresponding images obtained from his untreated sibling at the same age. (C) The comparison with an unrelated untreated patient with late-infantile MLD with similar age of onset, followed along disease course demonstrating white matter alterations (T2 hyperintensities) involving, in the earliest phases, periventricular areas and corpus callosum, spreading to subcortical regions, cerebellum and corticospinal tracts, and associated with progressive brain atrophy (enlargement of ventricular system and subarachnoid spaces). (D and E) Comparison of brain MRI total scores of patients with late-infantile (D) and early-juvenile by symptomatic status (E) treated with atidarsagene autotemcel [arsa-cel] versus natural history through an non-linear mixed-effects model based on a published methodology and strategy for model selection.^17^ MRI severity scoring system is a modified Loes’ score using methodology as previously described.^17^ The maximum total score is 31·5. MLD=metachromatic leukodystrophy.

Brain MRI total scores of patients with both late-infantile and early-juvenile disease at year 2 and 3 after treatment were significantly lower (less white matter involvement or less atrophy, or both) than in age-matched and disease subtype-matched NHx patients (appendix p 31). According to non-linear mixed-effects modelling, MRI total scores for treated patients with late-infantile and pre-symptomatic early-juvenile MLD stabilised at significantly lower levels throughout follow-up than they did for NHx patients, who displayed sharp increases indicating severe demyelination and atrophy (plateau parameter; p<0·0001 for late-infantile, p=0·0001 for pre-symptomatic early-juvenile; figure 4A–D). Similarly, MRI total scores of treated patients with early-symptomatic early-juvenile MLD, although stabilising at higher levels than for pre-symptomatic patients, were statistically significantly lower than in NHx patients (plateau parameter, p<0·0001; figure 4E).

Analyses of nerve conduction velocity index showed significant differences between treated patients with late-infantile MLD and NHx patients matched for age and disease subtype at year 2 (p=0·004) and year 3 (p=0·010; appendix p 32), whereas results in early-juvenile patients were more heterogeneous and showed marked imbalances at baseline than results for NHx patients (data not shown).

An exploratory sub-analysis of 12 treated patients with their untreated siblings confirmed the main treatment effects described above (appendix pp 27–30, 33–34), supporting the validity of the NHx cohort as a comparator.

## Discussion

Arsa-cel provided meaningful long-term clinical benefit in the most severe forms of MLD, confirming earlier preliminary findings.^17^ Arsa-cel therapy was generally well tolerated with no treatment-related serious adverse events; the most common adverse events were those expected in patients undergoing myeloablative conditioning and HSPC transplantation. There were no cases of transplant-related mortality, in contrast to known reactions with allogeneic haematopoietic stem-cell transplantation for MLD for which substantial mortality due to regimen-related toxicity, infections, or graft-versus-host disease has been reported.^11^, ^12^, ^23^

One patient died because of acute ischaemic stroke following an infectious event of unknown cause 13·6 months after treatment; after careful review of the available data, there was not sufficient information to establish a causal relationship and it was considered unlikely to be related to arsa-cel. Two patients with early-juvenile MLD, one with cognitive impairment at baseline and one whose gait progressively worsened between screening and infusion, had rapid disease progression shortly after treatment and died; both deaths were deemed unrelated to arsa-cel.

Polyclonal haematopoietic reconstitution of gene-corrected cells was observed in all patients, up to nearly complete gene-marked haematopoiesis, without signs suggestive of genotoxicity.

Busulfan-based, full-intensity myeloablative conditioning with pharmacokinetic-guided dosing is the current recommendation for lysosomal storage disorders.^24^, ^25^ We found no significant difference in terms of transduced cell engraftment or ARSA activity in PBMCs and cerebrospinal fluid between patients who received a standard myeloablative dose of busulfan and those who received submyeloablative conditioning. To maximise therapeutic benefit, we currently target a myeloablative dose of busulfan (AUC of 80 mg × h/L).

The transient occurrence of anti-ARSA antibodies was observed in a small number of patients and did not hamper the engraftment of transduced cells or reduce ARSA activity and had no obvious effect on clinical benefit or safety profile. Therefore, no additional immunosuppression during the conditioning regimen should be required. However, careful monitoring for possible immunogenicity is warranted to further investigate the extent of this adverse event, and immune-depleting treatment should be considered in case of associated clinically relevant manifestations.

Restoration of ARSA activity to normal levels in cerebrospinal fluid provides indirect evidence that a fraction of the infused genetically modified cells or their progeny migrated to the brain across the blood–brain barrier, engrafting and contributing to the local myeloid cell or microglial population in the brain. Similarly, migration of genetically modified blood cells to the peripheral nervous system and their possible differentiation into endoneural macrophages could have contributed to the treatment effects observed in nerve conduction velocity^26^, ^27^ by locally secreting functional ARSA. The therapeutic effect of lentiviral vector-HSPC gene therapy could be due to its ability to induce enzyme overexpression in HSPC progeny.^27^, ^28^ Reconstituted ARSA activity translated into prevention, stabilisation, or reduced rate of decline of motor skills and cognitive function and stabilisation of nervous system pathology compared with the natural course of disease. Treatment effects were particularly meaningful in the peripheral nervous system in patients with late-infantile MLD, which has been highly refractory to other therapeutic interventions.^11^

This work is based on a single-centre design using a historical control cohort followed up longitudinally with comparable assessments as the clinical study. This study design was considered the best option for a gene therapy treatment for a severe, ultra-rare paediatric disease with high unmet medical need in order to minimise variability.

Our results show that patients who were entering the rapid phase of disease progression at treatment could not benefit from treatment with arsa-cel. In addition to the two deaths, two patients (one with late-infantile disease and one with early-juvenile disease), displaying cognitive impairment at baseline,^17^ showed disease progression between enrolment and treatment, and had motor and cognitive deterioration at a rate similar to that for NHx patients after treatment. One additional late-infantile patient developed progressive motor and cognitive impairment, which was delayed compared with the disease course in untreated NHx patients at comparable chronological ages. At the time of treatment, the patient was judged pre-symptomatic but a retrospective review of pre-treatment acquisition of motor milestones showed a delay in the achievement of independent standing and walking, indicating that the patient was probably treated very close to the onset of overt disease manifestations. No differences were observed in quality attributes of the drug product, or in levels of engraftment or pharmacodynamic effects after treatment in these patients compared with the overall treated cohort.

On the basis of the clinical course of the two patients who showed disease progression between enrolment and treatment, inclusion criteria were refined during the clinical trial to avoid treatment of patients who were entering the rapid phase of disease progression at time of treatment while allowing treatment of early-juvenile patients displaying mild and stable symptoms after 6 months from onset; in late-infantile patients, rapid progression occurs almost immediately after symptom onset.^7^, ^8^ Since early-juvenile patients initially progress more slowly,^7^, ^8^ benefit is expected not only in pre-symptomatic patients but also in early-symptomatic patients.

To define the best window for gene therapy in patients with early-symptomatic early-juvenile disease, we did a retrospective analysis of baseline characteristics suggesting a further restriction of the treatment indication to patients without cognitive decline (IQ ≥85) and before loss of independent walking (gross motor function classification level 1 or less), without overt clinical deterioration between screening and infusion. Identification of additional laboratory, clinical, and instrumental biomarkers would also be highly relevant for predicting the treatment response. Ongoing long-term follow-up from this study and results from ongoing clinical trials of a cryopreserved formulation of arsa-cel in early-onset MLD (NCT03392987) and late-juvenile MLD (NCT04283227) will provide further evidence to support the MLD population most likely to benefit from treatment.

Given the variability in the time from initial symptoms to rapid progression, particularly in early-juvenile MLD, and the time required for engraftment and reconstitution of ARSA activity in PBMCs and cerebrospinal fluid following gene therapy (3–12 months), MLD patients must be diagnosed and treated expeditiously. All late-infantile and four early-juvenile patients were diagnosed early and enrolled in this study only because of an older affected sibling who, in most cases, could not be offered treatment because of advanced disease. This indicates the urgent need for newborn screening programmes for MLD. Of note, substantial advances in analytical testing in dried blood spots have been made, with several pilot screening studies underway.^29^, ^30^

An important limitation of our gene therapy approach is that most symptomatic patients will not be considered eligible for treatment because of rapid progression of the disease. Until the widespread implementation of newborn screening programmes, strategies using in-vivo injection of genetically-modified progenitors,^31^ lentiviral vectors,^32^ or adeno-associated virus vectors^33^ into the central nervous system could be explored with the goal of providing rapid supply of ARSA in patients who have entered the rapidly progressive phase of the disease. Another limitation of our study is the absence of a randomised comparator group. However, given the rarity and severity of the disease and the toxicity of the conditioning regimen, we considered placebo treatment unethical. Furthermore, the inclusion of untreated siblings with the same genotype in the NHx study and the stereotypical rapid disease progression observed in early-onset MLD patients in our NHx study^18^ and by others,^8^ especially in late-infantile patients,^4^ supports the NHx group as a robust comparator.

In conclusion, with up to 7·5 years of follow-up, these data confirm that arsa-cel provides meaningful clinical benefit to patients with early-onset MLD treated in pre-symptomatic and early-symptomatic stages of the disease, validating it as an effective therapeutic approach to alleviate the severe burden of MLD.^34^ The availability of this treatment could help to address the urgent unmet need in patients with this devastating disease.

## Data sharing

Because of the small number of participants in the study and potential for identification, individual patient data beyond what is included in the manuscript will not be available. The redacted study protocol will be available from the corresponding author upon request.

## Declaration of interests

FFu, VC, MGNS, AA, CB, FB, FFe, MM, FT, VG, SR, ESF, MPC, and MEB are investigators of gene therapy clinical trials for MLD sponsored by Orchard Therapeutics, the licence holder of investigational medicinal product arsa-cel. FFu and VC have acted as ad-hoc consultants for an Orchard Therapeutics advisory board. The MLD gene therapy was licensed to GlaxoSmithKline in 2014, and then to Orchard Therapeutics in 2018. Telethon and Ospedale San Raffaele are entitled to receive milestone payments and royalties for such a therapy. MSe and AB left San Raffaele Hospital and their role as principal investigators of the pivotal study on November, 2014, and September, 2015, respectively. AA subsequently became study principal investigator and responsible physician for treatment under expanded access frameworks. AB is currently a member of the scientific advisory board of Orchard Therapeutics and holds stock in Orchard Therapeutics. PMVR and CDS have a contract with Orchard Therapeutics to perform statistical analyses of gene therapy clinical trials for MLD. SMa and FM have a service contract with Ospedale San Raffaele. SMa has a contract with Orchard Therapeutics to perform ARSA activity on CSF in the clinical trial NCT03392987. JG-S, LCS, and GFD are former employees and hold stock in Orchard Therapeutics, which sponsored the clinical trial. All other authors declare that they have no financial interest related to the work described in the manuscript.
