# Supplementary material for: Lentiviral haematopoietic stem-cell gene therapy for early-onset metachromatic leukodystrophy: long-term results from a non-randomised, open-label, phase 1/2 trial and expanded access
Source: Lancet. 2022 Jan 22;399(10322):372–83. doi: 10.1016/S0140-6736(21)02017-1 (PMC8795071; doi:10.1016/S0140-6736(21)02017-1)
Supplement: Supplementary appendix [file mmc1.pdf]

THE LANCET

Supplementary appendix

This appendix formed part of the original submission and has been peer reviewed. We post it as supplied by the authors.

Supplement to: Fumagalli F, Calbi V, Natali Sora MG, et al. Lentiviral haematopoietic stem-cell gene therapy for early-onset metachromatic leukodystrophy: long-term results from a non-randomised, open-label, phase 1/2 trial and expanded access. *Lancet* 2022; **399:** 372–83.

Supplementary appendix

This appendix formed part of the original submission and has been peer reviewed.

We post it as supplied by the authors.

Supplement to: Fumagalli F, Calbi V, Natali Sora MG, et al. Lentiviral haematopoietic stem-cell gene therapy for early-onset metachromatic leukodystrophy: long-term results from a non-randomised, open-label, phase 1/2 trial and expanded access

Table of Contents

List of Investigators 3
Supplementary methods 4
- Pivotal study (201222): sample size 4
- Expanded-access frameworks 4
- Protocol deviations 4
- Outcome measures 5
- Intent-to-treat (ITT) population 5
- Matched analysis set 5
- Matched sibling analysis set 6
- Safety set 6
- Data cut-off 6
- GMFM and GMFC-MLD measures 6
- Statistical analyses for primary endpoints 7
- Statistical analyses for secondary and exploratory endpoints 7
- Analysis populations for GMFM scores and severe motor impairment-free survival 7
- Conditioning regimen 8

Supplementary results 8
- Overall survival 8
- Additional information regarding Patient 27 8
- Anti-ARSA antibodies 9
- Matched sibling analysis 9
- Table S1. Inclusion and exclusion criteria 10
- Table S2. Primary, secondary, and exploratory endpoints 12
- Table S3. Summary of drug product characteristics for arsa-cel 13
- Table S4. Grade 3 or higher adverse events reported in two or more patients (at least 7%) in the follow-up post-gene therapy phase in the post-GT by preferred term by study phase 15
- Table S5. Levels of gross motor function classification in MLD[2] 17
- Figure S1. Study schematic 17
- Figure S2. Patient disposition 18

Figure S3. Vector copy number in peripheral blood mononuclear cell and bone marrow subpopulations. Panel A shows myeloid-lineage cell subpopulations, panel B shows lymphoid-lineage cell subpopulations............ 19

Figure S4. Percentage of lentiviral vector-transduced cells in BM by drug product cell source 20

Figure S5. Vector copy number (VCN) by drug product cell source. Panel A shows VCN in BM-derived CD34$^+$ cells, panel B shows VCN in peripheral blood mononuclear cells 20

Figure S6. ARSA activity profiles in myeloid subpopulations of total peripheral blood mononuclear cells. Panel A shows CD14$^+$ and Panel B shows CD15$^+$ 22

Figure S7. Percentage of lentiviral vector-transduced cells in BM by conditioning regimen (sub-myeloablative or myeloabative) 23

Figure S8. Vector copy number in bone marrow-derived CD34$^+$ cells (Panel A) and peripheral blood mononuclear cells (Panel B) by conditioning regimen 24

Figure S9. ARSA activity profiles in bone marrow-derived mononuclear cells (Panel A) and peripheral blood mononuclear cells (Panel B) by conditioning regimen 25

Figure S10. Gross motor function measure total scores after 2 and 3 years in patients with pre-symptomatic (Panel A) and early-symptomatic (Panel B) early-juvenile MLD.. 26

Figure S11. Panel plots of gross motor function measure score profiles in arsa-cel treated patients with comparison to natural history data, including matched siblings (where applicable). Panel A shows late-infantile, Panel B shows early-juvenile 27

Figure S12. Panel plots of gross motor function classification levels by age in arsa-cel treated patients compared with natural history data, including matched siblings (where applicable). Panel A shows late-infantile, and Panel B shows early-juvenile 29

Figure S13. Treatment difference brain MRI scores at 2 and 3 years. Panel A shows late-infantile, and Panel B shows early-juvenile patients 31

Figure S14. Treatment difference NCV Index at 2 and 3 years for late-infantile patients 32

Figure S15. Cognitive age equivalent (performance) for treated patients compared with matched untreated siblings. Panel A shows late-infantile, and Panel B shows early-juvenile patients 33

Figure S16. Kaplan-Meier plot showing age at severe motor impairment or death in treated late-infantile (Panel A) or early-juvenile patients (Panel B) compared with matched untreated siblings 34

References 35

List of Investigators

**Clinical trial site:** IRCCS San Raffaele Scientific Institute, Milan, Italy	
San Raffaele Telethon Institute for Gene Therapy (SR-Tiget)	*Director*: Luigi Naldini<br>*Technicians*: Serena Acquati, Daniela Redaelli, Vanessa Attanasio, Simona Miglietta, Fabiola De Mattia<br>*Clinical Trial Office*: Marcella Facchini, Sara Locatelli, Ambra Corti, Stefano Zancan, Gigliola Antonioli, Giada Farinelli, Michela Gabaldo
Pediatric Immunohematology Unit and BMT Program	*Director:* Alessandro Aiuti<br>*Clinicians*: Francesca Fumagalli, Valeria Calbi, Maria Sessa, Federica Barzaghi, Francesca Ferrua, Maddalena Migliavacca, Francesca Tucci, Vera Gallo, Laura Lorioli, Salvatore Recupero, Elena Fratini, Maria Pia Cicalese, Maria Ester Bernardo, Alessandra Biffi<br>*Psychologists*: Francesca Ciotti, Maddalena Fraschini, Sabrina Canale<br>*Physiotherapist*: Marina Sarzana<br>*Research nurse*: Gigliola Antonioli
Unit of Hematology and Bone Marrow Transplantation	*Director:* Fabio Ciceri
Units of Neurology and Neurophysiology	*Director:* Massimo Filippi<br>*Clinicians:* Francesca Fumagalli, Maria Grazia Natali Sora, Maria Sessa, Alberto A Zambon, Ubaldo Del Carro
Neuroradiology Unit	Cristina Baldoli
Clinical Molecular Biology Laboratory	Ivana Spiga
Department of Anesthesia and Critical Care	Paolo Silvani, Maria Rosa Calvi
University Centre of Statistics in the Biomedical Sciences (CUSSB), Vita-Salute San Raffaele University	Paola M V Rancoita, Clelia Di Serio

Supplementary methods

Pivotal study (201222): sample size

The sample size of the pivotal trial (Study 201222) was progressively increased according to the following timelines:

- Protocol v. 1 (18-10-2009): Sample size, 8 patients (4 pre-symptomatic late-infantile, 4 pre- or early-symptomatic early juvenile)
- Protocol v. 3 (04-04-2011): Sample size, 8 patients (6 pre-symptomatic, comprising at least 4 late-infantile and 0–2 early-juvenile patients; and 2 early-symptomatic early-juvenile patients)
- Protocol v. 4 (10-01-2013): Sample size increased from 8 to 10 patients
- Protocol v. 6 (10-12-2013): Sample size increased from 10 to 14 patients
- Protocol v. 8 (03-10-2014): Sample size increased from 14 to 20 patients

The protocol version of Study 201222 that each patient was enrolled under is shown below:

Patient ID	Enrolled under protocol version
1, 2, 3, 4, 5, 6, 14	2.0
15	3.0
10	4.0
7	5.0
11, 16, 17	6.1
8	7.0
9, 12, 13, 18, 19, 20	8.0

The long period from first patient treated (i.e., 8 years) to this interim analysis is justified by the ultra-rare nature of the disease, the long follow-up period to evaluate the study endpoints, the progressive expansion of the sample size to strengthen the statistical analyses and the regulatory changes required throughout the clinical development of this therapy.

Expanded-access frameworks

Nine early-onset metachromatic leukodystrophy (MLD) patients treated under three separate expanded-access frameworks (EAFs) at the San Raffaele Telethon Institute for Gene Therapy (SR-Tiget) were included in this analysis. One patient (Patient 29) was treated under a nominal compassionate use scheme sponsored by Ospedale San Raffaele (OSR). Three patients were treated under the Italian law for "ATMP prepared on a non-routine basis" (Italian Hospital Exemption law; Patients 21–23) and five under another compassionate-use programme (CUP) (Patients 24–28) at OSR. The patients were treated according to the different EAFs due to the clinical study being closed for enrolment at the time. These programmes were conducted at the same clinical site and by the same study staff, using the same drug product and using similar enrolment criteria, study design, schedules of assessments, primary and secondary objectives, and endpoints as those in the primary clinical study. For the single patient CUP (Patient 29), information that required pre-approval (protocol, amendments, the informed consent, etc) were reviewed and approved by an institutional ethics committee in accordance with the International Conference on Harmonisation Good Clinical Practice (GCP) and applicable country-specific requirements. For the patients treated under the Hospital Exemption (HE) law (Patients 21–23), the Ethics Committee (EC) and the Italian Medicines Agency (AIFA) approved three separate HE protocols, one for each individual patient, between December 2015 and January 2016. Subsequently, a new CUP was initiated under the auspices of the Italian Ministerial Decree dated 7 September 2017, and five patients were treated (Patients 24–28). AIFA was notified of the opening of the CUP and also notified before treatment of each patient, and EC and AIFA approval documentation were retained at the treating clinical centre.

Protocol deviations

Study 201222 (Patients 1–20)

Protocol deviations defined as important per the Protocol Deviation Management Plan (GSK) were reported in treated subjects and included the following:

- Due to the long duration of study follow-up (up to 8 years) and the number of visits and assessments subjects were required to attend, 100% of subjects reported at least one protocol deviation pertaining to missed or out of window assessments. It was determined that none of these deviations affected subject safety or affected the veracity and integrity of the data.

- Informed consent/assent deviations (12 subjects, 60%). All of the important protocol deviations associated with informed consent forms (ICFs)/assent forms were related to parents signing an ICF in their non-native language (with the use of a translator) rather than a form written in the subject's native language.
- None of the protocol deviations were considered to affect data integrity or interpretationof results and none led to exclusion of subjects from the data analysis.

Three subjects had important protocol deviations related to study treatment:

- One subject (Subject 14; early-juvenile [EJ] subgroup) was treated with 2 drug products (DPs) due to a low yield of $CD34^+$ haematopoietic stem and progenitor cells (HSPCs) following the bone marrow (BM) harvest. The protocol was subsequently amended (in Protocol Amendment version 3.0) to allow for use of 2 DPs in the event of low yield from initial BM harvest.
- One subject (Subject 16; EJ subgroup) was treated with DP that, after administration, showed undefined particles in the syringe. The particulate was subsequently shown to consist predominantly of silicone oil from the syringe, with trace amounts of a secondary amide material. The local regulatory agency (AIFA) and the EC were notified of the finding and a corrective action was implemented to allow DP to be formulated into silicone-free 50 mL cryobags, rather than syringes. The subject did not experience any adverse events (AEs) considered related to arsa-cel.
- One subject (Subject 8; late-infantile [LI] subgroup) had a second BM back-up performed, which was noted as a protocol deviation. The second back-up was performed for the safety of the subject due to microbiological contamination of the first BM back-up that was therefore not compliant for infusion. This deviation did not have any impact on the DP manufacturing.

EAFs (Patients 21–29)

- At the time of the data cut-off for this interim clinical study report (CSR), no GCP compliance issues were identified by monitoring or audit.
- Of note, Patient 24 was the first patient enrolled in the CUP and the informed consent documentation was signed after the Screening visit date recorded in the electronic case report form (eCRF). Since the patient was affected with a LI variant of MLD and was identified very close to the expected age of onset according to her familial history, the patient was immediately evaluated by the site clinicians for possible eligibility for allogeneic haematopoietic stem cell transfer (HSCT) before the CUP was opened. Shortly after eligibility assessments for allogeneic HSCT were performed, the CUP protocol was approved by the EC and formally initiated. The family signed the ICF for arsa-cel treatment under CUP, and eligibility was reassessed for gene therapy arsa-cel. It should be noted that screening tests already conducted for the allogeneic HSCT eligibility assessments were not repeated in order to avoid subjecting the patient to repeat tests/assessments and to avoid further delay in treatment. The screening tests performed for the allogeneic HSCT eligibility assessment were recorded in the CUP eCRF.
- All nine patients had at least one missed assessment; these cases of non-compliance with study visit attendance or performance of individual study assessments limited the interpretation of some assessments.
- In the case of Patients 21 and 22, due to the exceptional nature of their circumstances, local hospital visits in the patient's country of residence were arranged on several separate occasions also prior to Month 36 in order to facilitate continued close monitoring of post-treatment safety and efficacy. In addition, Patient 26's Month 6 visit and Patient 27's Month 3 visit was conducted at local hospital in the country of residence.
- Under special circumstances, and at the responsible physician's discretion, long-term follow-up visits (Month 42 to Year 8) for both EAFs were allowed to be performed at a local hospital if the patient(s) were unable to travel to OSR for follow-up.

Outcome measures

A supplementary set of exploratory analyses were also specified for the integrated data set including severe motor impairment-free survival, cognitive performance and verbal age-equivalent scores, and analyses for magnetic resonance imaging (MRI) total scores based on non-linear mixed-effects models (Table S2).

Intent-to-treat (ITT) population

The ITT population included any patient enrolled into the primary study or EAFs who met the respective study inclusion criteria at screening, had signed an informed consent form, and received atidarsagene autotemcel (arsa-cel).

Matched analysis set

The matched analysis set included all patients from the ITT set and age- and disease subtype-matched untreated patients from the SR-Tiget natural history (NHx) study and was used for reporting efficacy and exploratory

efficacy endpoints related to gross motor function measure (GMFM), MRI, nerve conduction velocity (NCV), and neurological clinical evaluations at 2 and 3 years. The untreated subjects selected for the matched analysis were defined as subjects who:

- Are classified as late-infantile or early-juvenile MLD
- Had a study visit at which their chronological age fits within the range of ages for subjects treated with arsa-cel as described below.

For each of the late-infantile and early-juvenile subtypes in the ITT set at the 2- and 3-year analysis timepoints, the lower bound of the age range was based on the lowest age at the visit minus 3 months and the upper bound was the highest age at the visit. Additional subgroups of the matched analysis set were defined according to symptomatic status at the time of treatment.

NHx patients selected for the matched analysis were defined as LI or EJ, and had a study visit at which their chronological age fitted within the range of ages for treated patients with the same disease subtype at the time of analysis. If two or more visits were eligible for a single patient and both had non-missing GMFM scores, then the earliest visit was used for analysis at 2 years post-treatment, and the visit on or closest to 12 months after the visit used in the 2-year post-treatment analysis was used for the analysis at 3 years. All NHx patients were symptomatic at the time of enrolment but retrospective collection of data prior to enrolment enabled age-matched analysis with arsa-cel treated patients.

One patient (Patient 10) was classified as a clinical variant of intermediate severity between the classical LI and EJ forms. For the purposes of this analysis, this patient was included in the EJ cohort as this was the more conservative approach for evaluating the treatment effects of arsa-cel in this patient. More detail regarding this patient's classification was previously described in the Appendix of Sessa et al, 2016.[1]

Analysis was performed using the full matched analysis set, with no further matching performed within the set.

Matched sibling analysis set

The matched sibling analysis set included any patients in the ITT set who had untreated sibling(s) in the NHx study and the corresponding untreated sibling(s) from that study. This set was used for exploratory analysis to compare effects with untreated patients with a lower level of clinical variability in clinical phenotype and included 23 patients: 12 patients treated with arsa-cel and 11 in the NHx cohort. One patient from the NHx cohort was the sibling match for two treated patients in the primary study.

Safety set

This set included any enrolled arsa-cel treated patient across the primary study and EAFs. Two patients were screened in the primary study but did not receive treatment; one patient was withdrawn due to disease progression and the other withdrew consent and therefore did not receive treatment.

Data cut-off

Pivotal trial data cut-off was initiated when all patients had a minimum of 3 years of follow-up in the primary study. Data cut-off date for the primary analysis was 30 March 2018 and for the EAFs either 5 January 2018 (Patient 29) or 5 December 2018 (Patients 21–28).

GMFM and GMFC-MLD measures

A co-primary clinical efficacy endpoint was to assess an effect size of 10% difference in the total GMFM score in treated subjects at two years, as compared to a concurrent historical control group.

Gross motor function measure was used as primary endpoint since the beginning of the study in 2010 when gross motor function classification (GMFC-MLD) was not yet available. The GMFM instrument was originally developed as a measure of motor function changes over time in children with cerebral palsy,[2,3] but has also been used in patient groups with other disorders, such as Down syndrome[4] and osteogenesis imperfecta,[5] and in MLD.[6,7] A study conducted in patients with cerebral palsy, demonstrated that an increase between 7% and 24.6% of the GMFM score following rehabilitation therapies was rated as a medium to largely positive, clinically relevant motor improvement by blinded parents and physiotherapists.[3] Thus, an improvement of 10% was considered significant improvement in the quality of life of treated subjects compared to the natural course of the disease.

Following GMFC-MLD validation as a classification system for gross motor function in MLD,[8] GMFC-MLD was introduced in clinical development programme as a secondary efficacy assessment in April 2012 (Study 201222 protocol, version 3.0, 4 April 2020) and also retrospectively applied to all previous time points.

Statistical analyses for primary endpoints

A co-primary efficacy endpoint was the change from baseline in residual arylsulfatase A (ARSA) activity in peripheral blood mononuclear cells (PBMCs) at year 2 compared with pre-treatment values. The primary analysis conservatively imputed values at the lower limit of quantification (LLQ) (25·79 nmol/mg/h) for data below the LLQ of the assay, which was the case for most patients at Baseline in the integrated analysis. Increases in ARSA levels from Baseline were evaluated using a mixed- model repeated measures (MMRM) model.

One patient with late-infantile MLD in the ITT set did not contribute to the co-primary analysis as this patient did not have ARSA activity values in PBMCs recorded prior to treatment.

A type 1 error rate of 5% was preserved within each disease subtype using a sequential testing strategy with GMFM tested first and ARSA activity tested second, and interpretation was made using a stepwise approach. For GMFM, the null hypothesis was rejected with 95% confidence if the lower bound of the 95% confidence interval (CI) for the mean GMFM score difference (arsa-cel – NHx) was higher than 10%. For ARSA activity in PBMCs, the null hypothesis was rejected if the lower bound of the 95% CI for the ratio to baseline at Year 2 post-gene therapy (GT) was higher than 1.

Statistical analyses for secondary and exploratory endpoints

MRI total severity scores and NCV Index scores were analysed in a similar manner to the primary analysis of GMFM total score (%), evaluated at 2 years and 3 years post-treatment (analysis of covariance fitting age at MRI or NCV assessment and treatment arm [arsa-cel *vs* NHx] by disease subtype using the matched analysis set). Kaplan-Meier time-to-event curves were generated by disease subtype for survival analysis endpoints. Kaplan-Meier estimates of quartiles (median 25th and 75th percentiles) with two-sided, 95% CI were reported, if estimable. P values were calculated using an unstratified log-rank test. A generalisation of the log-rank test for interval-censored data to account for partial dates and periodic follow-up assessments was also calculated. Some longitudinal responses to outcome measures were observed to be non-linear in response shape. As such, post-hoc exploratory analyses were considered to better characterise the observed responses. Non-linear mixed effect models were therefore considered, using published methodology and strategy for model selection.[1]

Cognitive skills were prospectively assessed using the following neuropsychological tests both in Natural History and treated cohorts: Bayley Scale of Infant Development (BSID II and III) 1-3,[10-12] Wechsler Preschool and Primary Scale of Intelligence (WPPSI) (r and III edition),[13] Wechsler Intelligence Scale for children (WISC-III and IV),[14] according to patient's age.[15]

In order to describe the degree of cognitive and verbal impairment extending beneath the floor of 40 in some treated and NHx subjects and to record even minimal residual cognitive functions in severely impaired patients, the Bayley Scale was administered to older patients allowing quantification of an Age-Equivalent (mental developmental age). Developmental Quotient (DQ) scores obtained from Wechsler scales were transformed into an Age Equivalent generated by the following formula: DQ = (Age-Equivalent/Chronological Age) x 100[16] to allow intra and inter-subjects comparison.

DQ based on performance score (DQ_P) and based on language score (DQ_L) were grouped into the following categories representing the degrees of cognitive and verbal impairment: normal (DQ≥85), mild impairment (70≤DQ<85), moderate impairment (55<DQ<70) and severe impairment (DQ≤55).

Analysis populations for GMFM scores and severe motor impairment-free survival

Five patients with late-infantile MLD in the ITT set did not contribute GMFM data to the co-primary 2-year analysis as they either missed (n=1) or did not reach their 2-year GMFM assessment (n=4). Eight patients with late-infantile MLD in the NHx cohort were not included in the 2-year age-matched analysis of GMFM as their GMFM assessments were performed at an age above the upper limit used for this analysis. Three patients with early-juvenile MLD in the ITT set died before reaching their 2-year GMFM assessments and did not contribute to the co-primary endpoint analyses. One patient with early-juvenile MLD in the NHx cohort was not included in the 2-year age-matched analysis of GMFM as their GMFM assessments were performed at an age above the upper age limit used for this analysis. For the nine NHx patients (eight late-infantile and one early-juvenile) excluded from the co-primary GMFM analysis, GMFC-MLD levels were consistent with the loss of gross motor function (based on GMFM) in the patients from the NHx cohort included in the analysis, suggesting that the NHx patients included in the analysis are representative of the full NHx cohort.

Severe motor impairment free survival (sMFS) was defined as the interval from birth to loss of locomotion and sitting without support (GMFC-MLD level 5 or higher) or death from any cause; otherwise sMFS was censored at the last GMFC-MLD assessment date. All arsa-cel treated patients and NHx patients were included in this analysis.

Conditioning regimen

The conditioning regimen initially used consisted of 14 doses of busulfan (according to subject's weight), as a 2-hour intravenous (IV) infusion administered every 6 hours from Day −4 to Day −1.[16] Busulfan plasma levels were monitored by serial pharmacokinetic (PK) sampling and adjusted using a target dose area under the curve (AUC) of 4800 µg*h/L (range: 4200–5600 µg*h/L), which corresponds to an expected total cumulative AUC of 67 200 µg*h/L (range 58 800–78 400 µg*h/L). This conditioning regimen is referred to as sub-myeloablative (SMAC).

Subsequently, the conditioning regimen was modified from Protocol Version 6.1 (9 January 2014) with the goal of reducing the observed variability of transduced cell engraftment and was designed to produce a higher cumulative busulfan AUC. This is in line with emerging data available on the use of busulfan before allogenic hematopoietic cell transplantation.[17] This conditioning regimen consisted of body surface area-based dosing of busulfan according to the subject's age (80 mg/m^2/dose if ≤1 year of age; 120 mg/m^2/dose if >1 year) for a total of 4 doses, administered as a 3-hour IV infusion every 20–24 hours from Day −4 to Day −1. Busulfan plasma levels were monitored by timed PK sampling and adjusted using a target total cumulative AUC of 85 000 µg*h/L (range: 76 500–93 500 µg*h/L). This conditioning regimen is referred to as myeloablative (MAC).

The rationale for changing the conditioning regimen was based on preliminary analysis performed on the data from the first nine patients enrolled in Study 201222 and treated with the SMAC regimen. The busulfan exposure was increased to myeloablative concentrations to improve the therapeutic potential of arsa-cel on both the central and peripheral nervous systems (CNS; PNS) by reducing transduced cell engraftment variability and increasing engraftment levels. As such, the proposed modified conditioning regimen was aimed to increase Busulfan exposure by approximately 10% of the total AUC on average.

The protocol amendment allowed the investigators to reduce the busulfan target AUC to SMAC range when considering specific subject characteristics as risk factors such as, but not limited to, hepatic dysfunction, prematurity, thrombophilia, or very young age. Since the conditioning regimen was amended, additional patients received SMAC conditioning.

Overall, 13 subjects (45%) and 16 subjects (55%) were treated with a SMAC or MAC conditioning regimen respectively. The average, cumulative AUC in subjects who received a SMAC regimen was higher than expected (geometric mean 71 923·53 [95% CI 68 751·04–75 242·41]), but the average remained within the target range. The average, cumulative AUC in subjects who received a MAC regimen was consistent with the target AUC (geometric mean 84 043·08 [95% CI 82 369·52–85 750·65]).

Supplementary results

Overall survival

At the time of data cut-off for the integrated analysis, overall survival (OS) in treated late-infantile MLD patients (n=16) was 100% with a median follow-up time of 3·0 years (1·0–7·5). Four of these patients had more than 6 years of follow-up and two more than 7 years, with the first treated patient reaching an age of 8.7 years. Among the 19 NHx patients, five had died by the age of 6 years, corresponding to an estimated OS of 70·8%, while seven treated LI MLD patients had survived to that age. Comparable OS was seen in the treated and NHx EJ patients where at a chronological age of 9 years, estimated OS was 75·5% and 100%, respectively. At 10 and 11 years of age, an estimated 75·5% of treated EJ patients were alive compared with 88·9% and 76·2%, respectively, of NHx patients.

Additional information regarding Patient 27

One patient in the CUP programme (Patient 27) died after treatment with arsa-cel on Day 415 (13·6 months). The patient was pre-symptomatic (EJ variant) at the time of treatment and remained asymptomatic at the time of experiencing an ischemic cerebral infarction (left hemisphere cerebral ischaemic stroke) on Day 414 that led to death (Grade 5) on Day 415.

Routine follow-up for the patient performed at the Year 1 study visit prior to the serious AE of ischaemic cerebral infarction showed that the patient was clinically well. The patient had no known predisposing risk factors for a thrombotic event, no vascular/endothelial complications after treatment, and no known history of trauma in the days leading up to the event. From a hematological point of view, blood counts were within normal range in all subpopulations and bone marrow aspirate was normal. CSF analysis was normal. Engraftment levels of transduced cells were in keeping with previous follow up and in the range observed in other patients.

At event onset, the patient was reported to have a recent history of coryzal symptoms (runny nose) for some days, complained of a headache, was sleepy and experienced suspected seizures. The patient was intubated and treated for status epilepticus. Computed tomography (CT) scan with angiography revealed cerebral oedema and ischaemic

cerebral infarction and the patient died on the same day (Day 415) due to the event of ischaemic cerebral infarction. The cause of the event was unknown, but the investigator assessed the event of ischaemic cerebral infarction as not related to arsa-cel, stating there was not sufficient information to establish a causal relationship between the event and gene therapy. The family declined post-mortem examination. The site continued to investigate the event in depth to define the exact nature, cause and pathophysiology of this acute event. The integration site analyses performed up to the latest follow-up by SR-TIGET showed highly polyclonal vector integration and did not reveal signs of clonal expansion or clonal dominance.

The conclusion from regulatory authorities was that a relationship between the ischaemic event and gene therapy appears unlikely as the event took place approximately a year post gene therapy. Notably, the subject was responsive to arsa-cel treatment. Further information related to the case suggests that an infection is likely the cause of the event; however, it was not possible to determine whether this had a bacterial or viral origin.

Anti-ARSA antibodies

Five AEs of presence of anti-ARSA antibodies occurred in four patients (Patients 21, 22, 25, and 28). In all four patients the event started within 1 year post gene therapy and in two of them followed other auto-antibody positivity (anti-platelet antibodies and Coombs positivity), which can occur in the early phase post-transplant. In three of four patients the onset of the anti-ARSA antibodies followed veno-occlusive disease and/or thrombotic microangiopathy (TMA) as transplant complications.

All patients' tests had resolved to negative test results, either spontaneously (n=1, Patient 28) or after one cycle of rituximab (n=3).

In two cases (Patients 21 and 22) patients received B-cell depletion therapy (rituximab) for other clinical reasons (auto-immune cytopenia, post-transplant TMA).

In two cases (Patients 25 and 28) B-cell depletion with rituximab was started by investigators since there was a temporal relationship between the detection of anti-ARSA antibodies and the initial report of gait disturbance as AEs. A sample was taken from Patient 28 for a repeated test for anti-ARSA antibodies on the same day that the patient received the first dose of rituximab; the test eventually turned out negative, indicating a spontaneous clearance of antibodies. No firm conclusions can be drawn considering the very low titres of anti-ARSA antibodies in this subject, the lack of impact on pharmacodynamic outcomes, and the persistence of motor disfunction well after antibodies turned negative.

Matched sibling analysis

An efficacy sub-analysis was conducted on 12 treated patients compared with their corresponding untreated siblings. The matched sibling analysis showed treatment effects on gross motor function (figures S6–S7) and cognition (figure S10), comparable with those observed between the ITT set and NHx cohorts, supporting the validity of the overall NHx cohort as a comparator.

Table S1. Inclusion and exclusion criteria

**Inclusion criteria***
Confirmed diagnosis of MLD based on:<br>• ARSA activity below the normal range in peripheral blood mononuclear cells or fibroblasts<br>• Presence of two-disease causing mutations of either known or novel alleles (to exclude polymorphisms from novel alleles, searches of polymorphism databases and screening of at least 100 chromosomes from non-MLD patients were conducted)<br>• Presence of sulfatides in a 24-hour urine collection to exclude MLD carriers and patients with ARSA pseudodeficiency
Pre-symptomatic late-infantile patients
Pre- or early-symptomatic early-juvenile patients
**Late-infantile**<br>Two out of three criteria to be met<br>• Age at onset of symptoms in the older sibling(s) ≤30 months<br>• And/or two null (0) mutant ARSA alleles<br>• And/or peripheral neuropathy at electroneurographic study
**Early-juvenile**<br>Two out of three criteria to be met<br>• Age at onset of symptoms (in the subject or in the older sibling) between 30 months and 6 years (had not celebrated their 7th birthday)<br>• And/or one null (0) and one residual (R) mutant ARSA allele(s)<br>• And/or peripheral neuropathy at electroneurographic study
Pre-symptomatic clinical status was defined as patients without neurological impairment (disease-related symptoms), with or without signs of the disease revealed by instrumental evaluations (electroneurographic and brain magnetic resonance imaging). In October 2014 (Protocol 8.0, 3 Oct 2014), an additional step of eligibility confirmation was introduced for pre-symptomatic subjects to avoid treatment of late-infantile patients developing first symptoms of MLD between the screening visit and cell collection for IMP manufacturing and the start of the conditioning regimen
Early-symptomatic clinical status (for the early-juvenile variant) was initially defined as subject identified within 6 months from the first reported symptoms (two early-juvenile patients were enrolled using this definition: MLD04 under Protocol 2·0, 26 Jan 2010 and MLD08 under Protocol 3·0, 4 Apr 2012).<br>Subsequently, early-symptomatic early-juvenile patients were defined as patients meeting the following two criteria:<br>• Intelligence quotient ≥70, and<br>• The ability to walk independently for ≥10 steps (ie, in Protocol 6·0, 10 Dec 2013)<br>The rationale for the inclusion criteria change was to prevent enrolment of severely impaired patients or patients who had entered a rapid phase of disease progression for whom benefit from treatment was not expected, and to allow treatment of early-symptomatic early-juvenile patients who were mildly symptomatic after 6 months, based on emerging data from the TIGET natural history study and data published in Kehrer et al. 2011[9] that the rapid phase of decline starts when affected subjects lose the ability to walk. An additional step of eligibility confirmation was added (Protocol 6.0, 10 Dec 2013) prior to cell collection for IMP manufacturing and the start of the conditioning regimen in order to exclude early-symptomatic subjects showing worsening symptoms between enrolment and treatment. Specifically, the principal investigators might not confirm treatment indication if disease progression, tested with neurological and neuropsychological evaluations, between enrolment and the scheduled treatment was rapid and severe
Parental/guardian/patient signed informed consent
**Exclusion criteria**
A positive test result for HIV; hepatitis C or B
Affected by neoplastic diseases
Cytogenetic alterations typical of myelodysplastic syndrome or acute myelogenous leukaemia
End-organ functions or any other severe disease which, as judged by the investigator, would make the patient inappropriate for study entry
Enrolment in other trials
Had undergone allogeneic HSCT in the previous 6 months
Had undergone allogeneic HSCT with evidence of residual cells of donor origin

*The inclusion criteria for patients treated under Hospital Exemption (HE; Patients 21–23) and OSR Compassionate Use Program (CUP; Patients 24–28) differed from the criteria used in the clinical trial in that only pre-symptomatic patients were allowed to be enrolled. The HE included only pre-symptomatic late-infantile patients, with the same definition of pre-symptomatic status as the clinical trial. For the CUP, patients were classified as pre-symptomatic if they a) did not have a delay in the achievement of independent walking or standing with abnormal signs at neurological evaluation, or b) did not have documented neurological symptoms of MLD associated with cognitive, motor, or behavioural functional impairment or regression. For the CUP only, the inclusion criteria allowed enrolment of patients with an older sibling index case of late-infantile or early-juvenile MLD as defined above, or with an intermediate phenotype based on symptom onset in the older siblings of ≤6 years of age but inability to unambiguously classify the index case as late-infantile or early-juvenile; or in those without an older affected sibling, the totality of the data available to the treating physician strongly suggested that the patient had an early onset variant of MLD (≤6 years of age) likely to benefit from treatment with approval by the medical monitor.

Patient 29 was an early-symptomatic early-juvenile MLD patient treated under a nominal compassionate use scheme as the study had closed to EJ patients at the time of Patient's 29 treatment. While there were no formal inclusion or exclusion criteria established, the patient met all eligibility criteria under the clinical trial protocol with the exception that the patient was symptomatic for 8 months, which exceeded the inclusion requirement for early-juvenile patients (symptomatic for ≤6 months) in the protocol version (v 4·0) active at the time of Patient 29's treatment.

ARSA=arylsulfatase A. HCT=hematopoietic stem-cell transplantation. MLD=metachromatic leukodystrophy.

Table S2. Primary, secondary, and exploratory endpoints

The efficacy and safety co-primary and secondary endpoints evaluated in the clinical development programme for arsa-cel were similar across all clinical studies and EAFs. Analyses of additional exploratory endpoints included in table S2 were specified for the integrated analysis.

**Co-primary efficacy endpoints**
• An improvement of >10% in the total GMFM score in treated patients, when compared with total GMFM scores from the NHx study patients evaluated at Year 2 post-treatment<br>• Change from baseline of ARSA activity in total PBMC at Year 2 post-treatment compared with pre-treatment values
**Secondary efficacy endpoints**
• An improvement of >10% in the total GMFM score in treated patients evaluated at Year 3 post-treatment, when compared with total GMFM scores from the NHx study patients<br>• GMFC-MLD levels at different ages in treated patients compared with the NHx study patients<br>• Brain MRI total score at Years 2 and 3 post-treatment compared with the NHx study patients<br>• NCV index at Years 2 and 3 post-treatment compared with the NHx study patients, including evaluation in individual sensory and motor nerves<br>• An engraftment of the transduced cells above 4% in bone marrow-derived clonogenic progenitor cells[18] at 12 months after the transplant. VCN was also evaluated in total PBMCs, total BM, and peripheral blood and BM cell subpopulations<br>• Correlations between transduced cell engraftment parameters and busulfan exposure including evaluations of percentage lentiviral-positive cells, VCN in total PBMCs and BM mononuclear cells) at 12 months and busulfan exposure (ie, total area under the curve for concentration-time) during the conditioning phase<br>• The measurement of Intelligence Quotient (IQ) values above 55 at 24, 30, and 36 months after treatment<br>• Age at death, defined as the interval from birth to the event of death from any cause, otherwise patients were censored at the last contact date up to and including the data cut-off date<br>• Change from baseline of ARSA activity in mononuclear cells and other cell types compared with pre-treatment values in BM mononuclear cells and peripheral blood and BM subpopulations at Year 2 post-treatment. ARSA activity was also measured in cerebrospinal fluid at multiple visits
**Safety endpoints**
• Absence of engraftment failure or delayed haematological reconstitution, defined as absolute neutrophil count 500/µL at +60 days after transplantation, with no evidence of BM recovery, requiring cellular back-up administration<br>• Absence of conditioning regimen related toxicity determined by surveillance of clinical (National Cancer Institute [NCI] ≥2) and laboratory (NCI ≥3) parameters that will be applied in the short- and long-term follow-up of the treated subjects in order to assess the degree of morbidity associated with the conditioning regimen<br>• Safety and tolerability of lentiviral-transduced cell infusion<br>o The short-term safety and tolerability of lentiviral-transduced cell infusion was evaluated on the basis of AEs reporting and monitoring of the systemic reactions to cell infusion (eg, fever, tachycardia, nausea and vomiting, joint pain, skin rash, etc.). The short-term safety of lentiviral transduced cell infusion consists of the absence of serious adverse events within 48 hours from infusion<br>o The long-term safety of lentiviral-transduced cell infusion was evaluated as the absence of replication competent lentivirus (RCL) and the absence of abnormal clonal proliferation (ACP): Abnormal haematopoietic clonal expansion was monitored by clinical and laboratory surveillance, TCR Vbeta repertoire study, and bone marrow examination<br>• Monitoring of AEs and SAEs, routine laboratory tests, vital signs, physical examinations, specialist examinations, and diagnostic imaging and instrumental tests<br>• Absence of immune responses against the transgene (anti-ARSA antibodies)
**Exploratory quality-adjusted survival-based endpoints**
• Age at severe motor impairment or death – severe motor impairment-free survival, defined as the interval from birth to the earlier of loss of locomotion and sitting without support (GMFC level 5 or higher) or death from any cause; otherwise patients were censored at the last GMFC assessment date<br>• Time from disease onset to severe motor impairment or death (in early-symptomatic early-juvenile patients only), defined as the interval from disease onset to the earlier of loss of locomotion and sitting without support (GMFC level 5 or higher) or death from any cause; otherwise, patients were censored at the last GMFC assessment date
**Other exploratory endpoints**
• Development quotient and age-equivalent profiles to assess acquisition or maintenance of cognitive skills<br>• MRI total score mean response profiles based on non-linear mixed effects (NLME) models

AE=adverse event. ARSA=arylsulfatase A. BM=bone marrow. GMFC=gross motor function classification. GMFM=gross motor function measure. MLD=metachromatic leukodystrophy. MRI=magnetic resonance imaging. NHx=natural history. PBMC=peripheral blood mononuclear cells. SAE=serious adverse event. SD=standard deviation. SR-TIGET=San Raffaele Telethon Institute for Gene Therapy. VCN=vector copy number.

Table S3. Summary of drug product characteristics for arsa-cel

		Bone marrow (N=26)		BM+MPB (N=2)		MPB (N=1)	Overall (N=29)*	
**Parameter**		**LI (N=16) n (%)**	**EJ (N=10) n (%)**	**Patients 14 & 29 (EJ), BM Source (N=2)** n (%)**	**Patients 14 & 29 (EJ), MPB source (N=2)** n (%)**	**Patient 27 (EJ) (N=1) n (%)**	**LI (N=16) n (%)**	**EJ (N=13) n (%)**
**Number of nucleated cells (x10^6/kg)**	Geometric mean	14·21	10·70	5·87	5·10	17·9	14·21	11·24
	% CVb	44·1	29·7	50·1	28·1	–	44·1	29·7
	95% CI	11·35–17·79	8·69–13·17	N/A	N/A	–	11·35–17·79	9·42–13·39
	Median	15·15	11·35	6·20	5·20	–	15·15	11·50
	Min, max	6·9, 26·9	7·4, 18·0	4·2, 8·2	4.2, 6.2	–	6·9, 26·9	7·4, 18·0
**Number of CD34$^+$ HSPCs (x10^6/kg)**	Geometric mean	12·01	8·76	5·05	5·06	17·6	12·01	9·49
	% CVb	51·1	30·7	41·8	26·9	–	51·1	33·5
	95% CI	9·29–15·53	7·06–10·85	N/A	N/A	–	9·29–15·53	7·79–11·55
	Median	13·10	8·95	5·25	5·15	–	13·10	9·70
	Min, max	4·2, 25·9	6·0, 16·3	3·8, 6·7	4·2, 6·1	–	4·2, 25·9	6·0,17·6
**Number of CFU-GM (/10^6 cells)**	Geometric mean	35 690·6	60 417·2	43 205·9	92 281·2	33 700·0	35 690·6	58 796·1
	% CVb	55	26	64	31	–	55	30
	95% CI	27 099·8–47 004·8	50 450·8–72 352·3	N/A	N/A	–	27 099·8–47 004·8	49 377·2–70 011·7
	Median	31 250·0	62 350·0	47 000·0	94 350·0	–	31 250·0	62 000·0
	Min, max	19 000, 86 000	33 800, 85 300	28 500, 65 500	74 700, 114 000	–	19 000, 86 000	33 700, 85 300
**Transduction efficiency (CFU-C) (%)**	Geometric mean	91·72	88·67	88·09	84·44	81·0	91·72	87·69
	% CVb	9·3	16·1	13·7	13·1	–	9·3	14·7

	95% CI	87·31–96·35	79·06–99·45	N/A	N/A	–	87·31–96·35	80·30–95·77
	Median	94·50	94·00	88·50	84·80	–	94·50	94·00
	Min, max	68·0, 100·0	61·0, 100·0	80·0, 97·0	77·0, 92·6	–	68·0, 100·0	61·0, 100·0
**Vector copy number (VCN/cell)**	Geometric mean	4·597	3·633	2·755	1·378	3·40	4·597	3·332
	% CVb	49·26	53·83	25·95	47·83	–	49·26	51·24
	95% CI	3·586–5·893	2·533–5·212	N/A	N/A	–	3·586–5·893	2·489–4·461
	Median	4·600	3·017	2·800	1·450	–	4·600	2·934
	Min, max	1·72, 8·70	1·70, 9·40	2·30, 3·30	1·00, 1·90	–	1·72, 8·70	1·70, 9·40

Total volume infused (mL) for the overall cohort (n=29) was a geometric mean of 23·12 (range: 19·6–40·0; median: 20·00; 95% CI 21·08–25·36). Total nucleated cells infused (10^6 cells) for the overall cohort (n=29) was a geometric mean of 159·0 (range: 62–297; median: 181·0; 95% CI 136·0–186·0). Percentage cell viability for the overall cohort (n=29) was a geometric mean of 99·04% (range: 98·0–100·0; median: 99·00%; 95% CI 98·82–99·27). 95% CIs have been presented when there are 3 or more subjects with available data. *For the integrated analysis, the summary of drug product has been combined across different cellular sources (BM harvest or PB collection). The drug product characteristics reported in the registrational study summarises separately by cellular source. **Patient 14 and Patient 29.

BM=bone marrow. CFU-C=colony-forming units in culture. CFU-GM=colony-forming units in culture-granulocyte monocyte. CI=confidence interval. CVb=coefficient of variation between patients. EJ=early-juvenile. geo=geometric. HSPCs=haematopoietic stem and progenitor cells. LI=late-infantile. MPB=mobilised peripheral blood. VCN=vector copy number. N/A, Not applicable

Table S4. Grade 3 or higher adverse events reported in two or more patients (at least 7%) in the follow-up post-gene therapy phase in the post-GT by preferred term by study phase

Preferred term	Pre-tx	Tx	Acute	3 months post-tx	Short term	Long term	Total follow-up post-GT
	(N=29) n (%)	(N=29) n (%)	(N=29) n (%)	(N=29) n (%)	(N=29) n (%)	(N=16) n (%)	(N=29) n (%)
Any event	7 (24)	8 (28)	0	27 (93)	22 (76)	5 (31)	29 (100)
Febrile neutropenia	0	0	0	23 (79)	0	0	23 (79)
Gait disturbances*	1 (3)	0	0	5 (17)	9 (31)	1 (6)	15 (52)
Stomatitis	0	0	0	12 (41)	0	0	12 (41)
Motor dysfunction*	0	0	0	2 (7)	7 (24)	0	9 (31)
Muscle spasticity*	0	0	0	1 (3)	6 (21)	2 (13)	9 (31)
Mucosal inflammation	0	0	0	9 (31)	0	0	9 (31)
Aphasia*	0	0	0	1 (3)	4 (14)	1 (6)	6 (21)
Ataxia*	0	0	0	2 (7)	3 (10)	0	5 (17)
Device-related infection	3 (10)	0	0	2 (7)	3 (10)	0	5 (17)
Neutropenia†	0	0	0	5 (17)	0	0	5 (17)
Cognitive disorder*	0	0	0	0	3 (10)	1 (6)	4 (14)
Dysarthria*	0	0	0	1 (3)	4 (14)	0	5 (17)
Dysphagia*	0	0	0	0	3 (10)	1 (6)	4 (14)
Vomiting	0	0	0	3 (10)	0	1 (6)	4 (14)
Enteritis	0	0	0	0	3 (10)	0	3 (10)

Preferred term	Pre-tx	Tx	Acute	3 months post-tx	Short term	Long term	Total follow-up post-GT
	**(N=29) n (%)**	**(N=29) n (%)**	**(N=29) n (%)**	**(N=29) n (%)**	**(N=29) n (%)**	**(N=16) n (%)**	**(N=29) n (%)**
Metabolic acidosis	2 (7)	4 (14)	0	2 (7)	1 (3)	0	3 (10)
Pneumonia	0	0	0	1 (3)	2 (7)	1 (6)	3 (10)
Veno-occlusive disease	0	0	0	3 (10)	0	0	3 (10)
Atypical haemolytic uraemic syndrome	0	0	0	2 (7)	0	0	2 (7)
Clostridium difficile colitis	0	0	0	2 (7)	0	0	2 (7)
Epistaxis	0	0	0	2 (7)	0	0	2 (7)
Rash erythematous	0	0	0	2 (7)	0	0	2 (7)
Seizure*	0	0	0	0	1 (3)	1 (6)	2 (7)

*AEs were manually reviewed by the sponsor and confirmed by the investigators after database lock to identity AEs typically associated with MLD symptoms; the decision to classify an event as associated with MLD was based on clinical judgment and experience with MLD. †Prolonged neutropenia (neutropenia beyond day 45 post-treatment).

AE=adverse event. GT=gene therapy. Tx=treatment.

Table S5. Levels of gross motor function classification in MLD[2]

Level	Definition
0	Walking without support with quality of performance normal for age
1	Walking without support but with reduced quality of performance, ie, instability when standing or walking
2	Walking with support. Walking without support not possible (fewer than 5 steps)
3	Sitting without support **and** locomotion such as crawling or rolling. Walking with or without support not possible
4	Sitting without support but no locomotion, **or** sitting without support not possible but locomotion such as crawling or rolling
5	No locomotion nor sitting without support, but head control is possible
6	Loss of any locomotion as well as loss of any head and trunk control

Figure S1. Study schematic

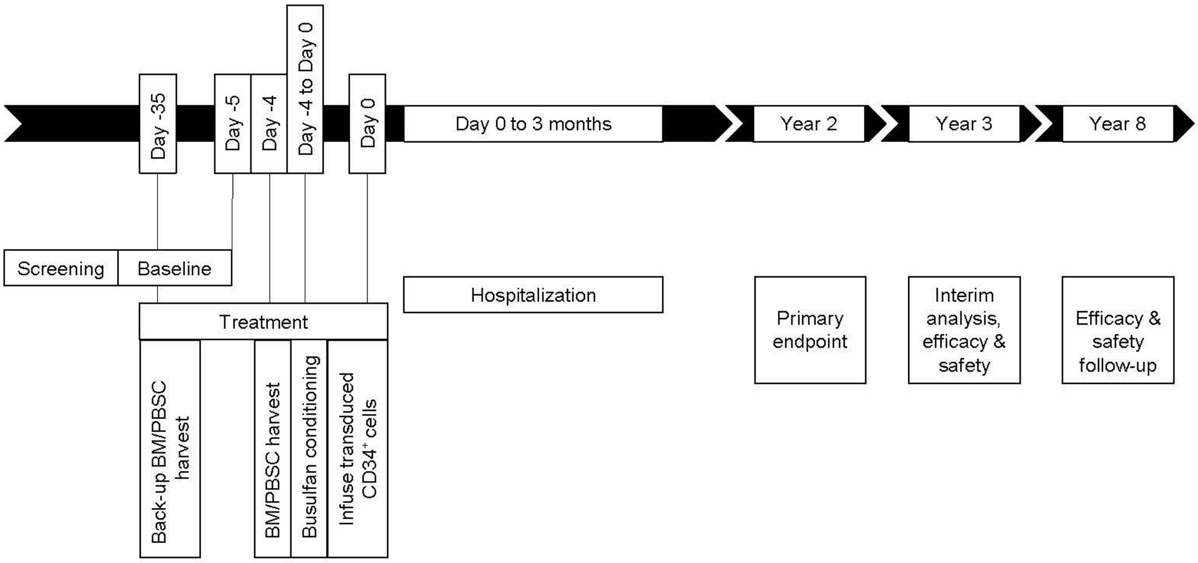

Figure S2. Patient disposition

Panel A: Intent-to-treat population

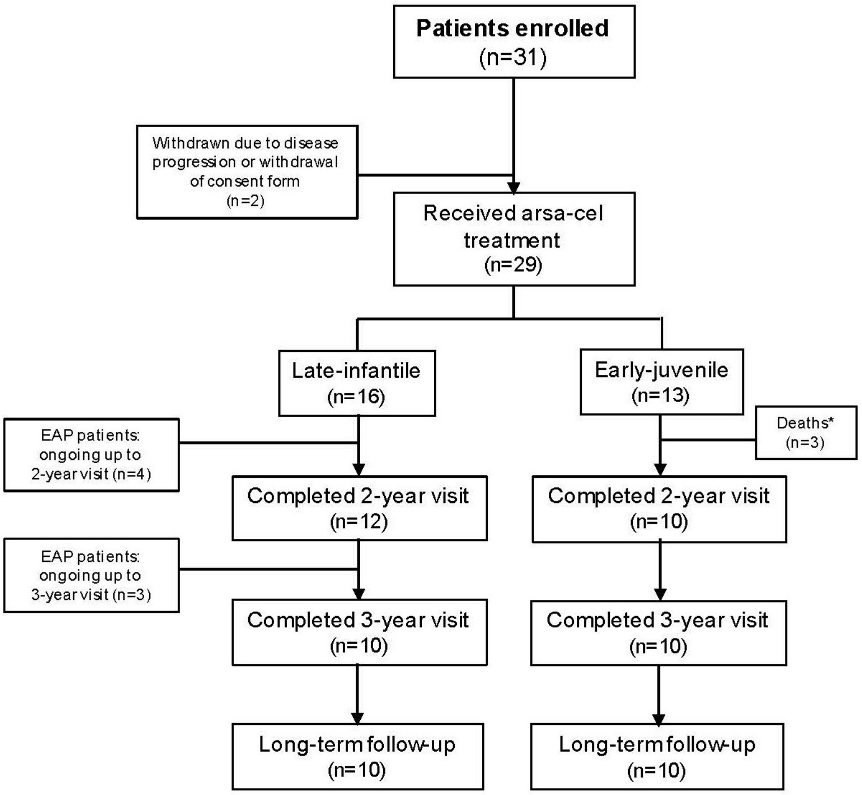

*Two deaths due to disease progression, one death due to cerebral ischaemic infarct unrelated to treatment
EAP, early access programme

Panel B: SR-Tiget NHx study population

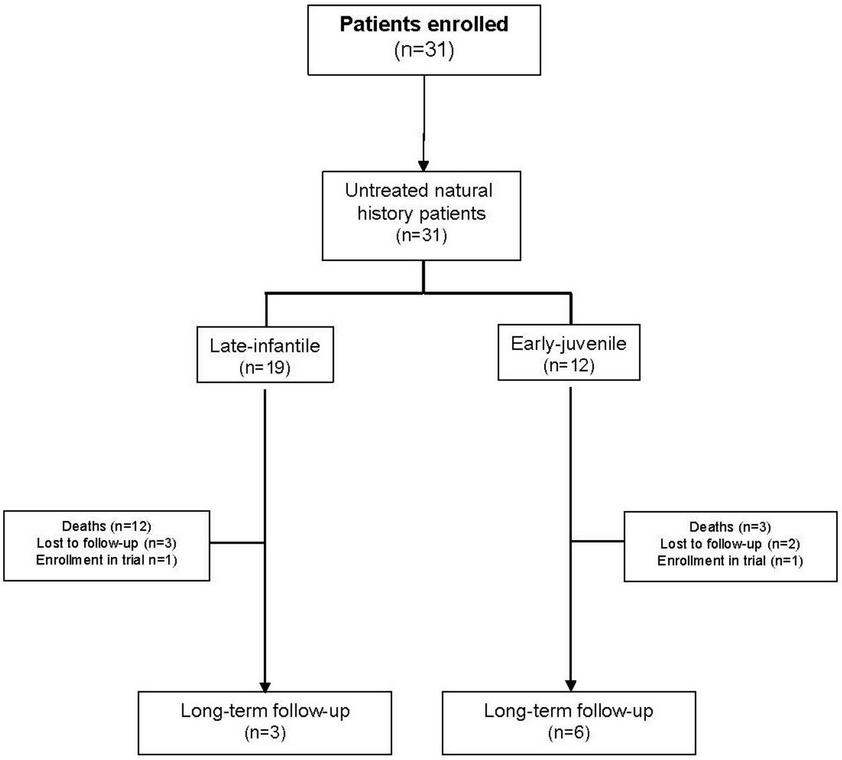

Figure S3. Vector copy number in peripheral blood mononuclear cell and bone marrow subpopulations. Panel A shows myeloid-lineage cell subpopulations, panel B shows lymphoid-lineage cell subpopulations

A

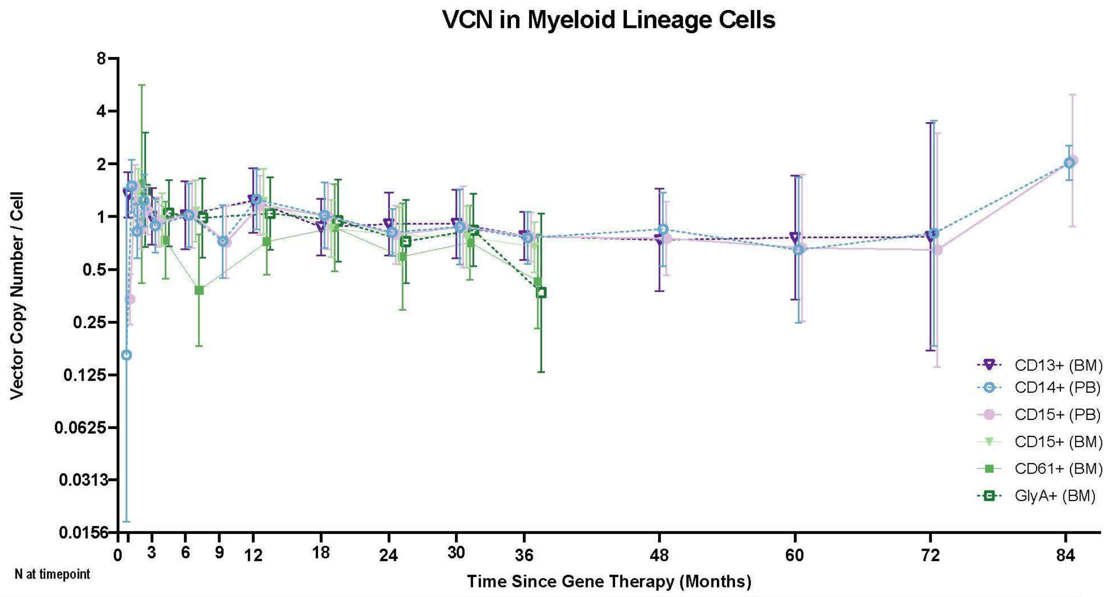

N at timepoint													
CD13+ (BM) N	25	27	24		24	18	19	15	15	10	7	4	
CD14+ (PB) N	24	28	23	20	27	21	21	17	19	12	7	4	3
CD15+ (PB) N	23	28	24	20	27	20	21	18	19	12	7	4	3
CD15+ (BM) N	21	27	24		24	17	19	16	15				
CD61+ (BM) N	4	13	13		16	12	9	11	9				
GlyA+ (BM) N	10	17	19		18	12	12	12	10				

B

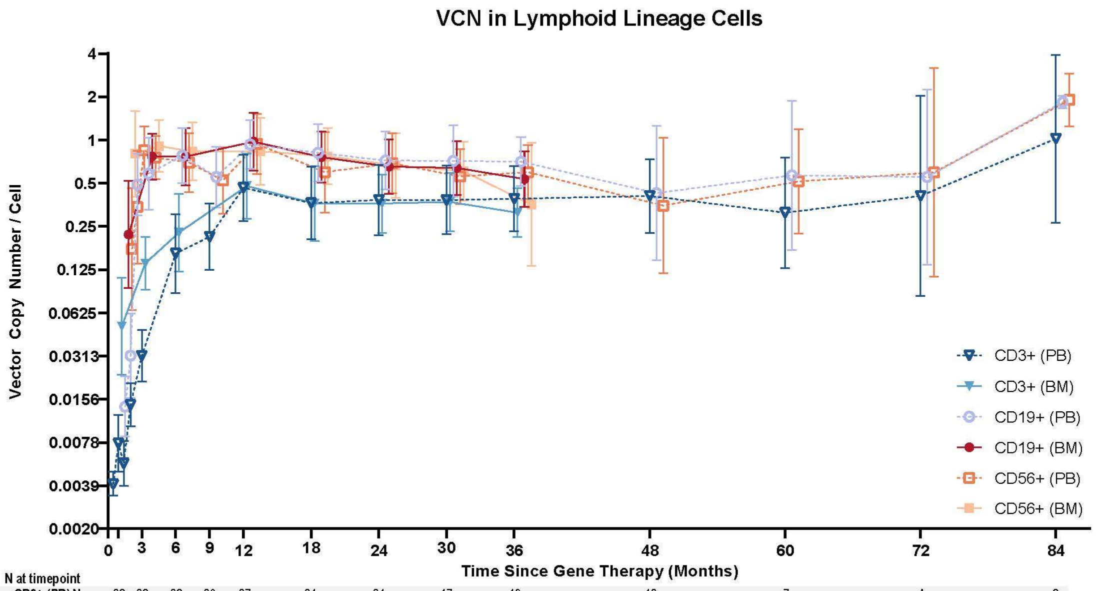

N at timepoint													
CD3+ (PB) N	23	28	23	20	27	21	21	17	19	12	7	4	3
CD3+ (BM) N	14	26	23		24	18	19	15	15				
CD19+ (PB) N	16	25	23	20	26	21	21	16	19	12	7	4	3
CD19+ (BM) N	14	26	24		24	18	19	16	15				
CD56+ (PB) N	15	26	22	20	26	21	19	16	19	12	7	4	3
CD56+ (BM) N	9	22	23		21	15	18	15	13				

Limit of quantification (LOQ) is 0·0037 VCN/cell. Values < LOQ are imputed at LOQ. Data points are geometric means with associated 95% confidence intervals and are presented at timepoints where there were at least three subjects with non-missing data. BM=bone marrow. PB=peripheral blood.

Figure S4. Percentage of lentiviral vector-transduced cells in BM by drug product cell source

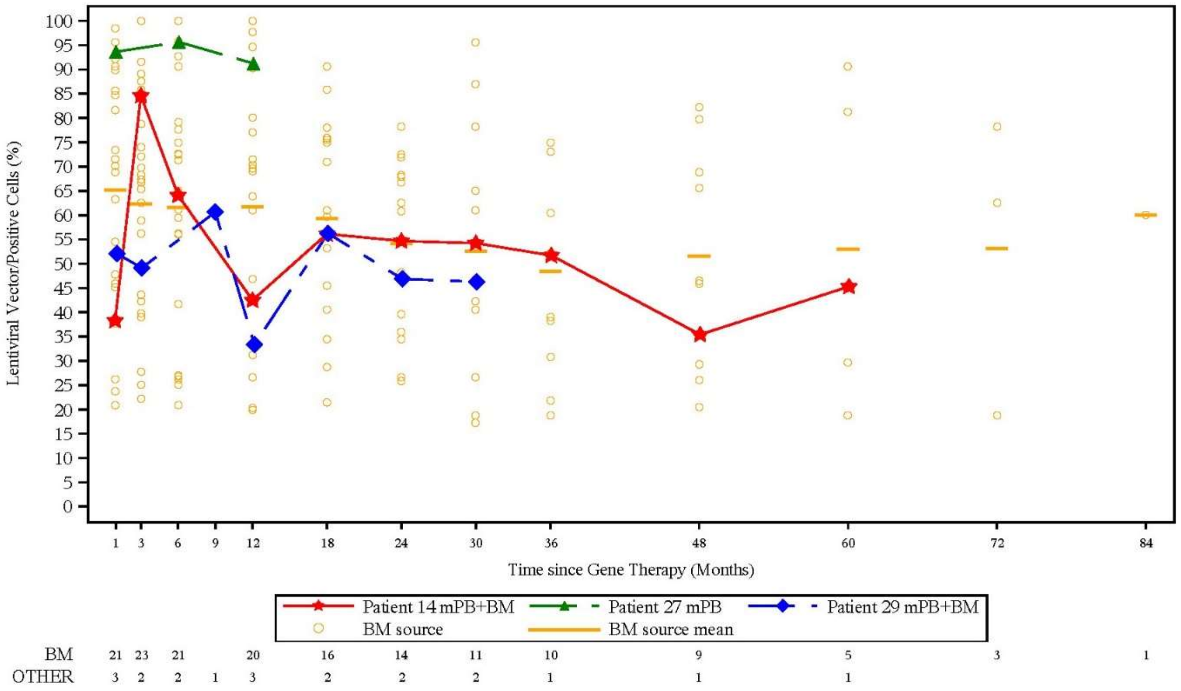

Data from patients 14, 27, and 29 is overlaid on the range of data from patients who received BM-derived $CD34^{+}$ cells as the sole cell source to manufacture the drug product. BM=bone marrow; mPB=mobilised peripheral blood.

Figure S5. Vector copy number (VCN) by drug product cell source. Panel A shows VCN in BM-derived $CD34^{+}$ cells, panel B shows VCN in peripheral blood mononuclear cells

A

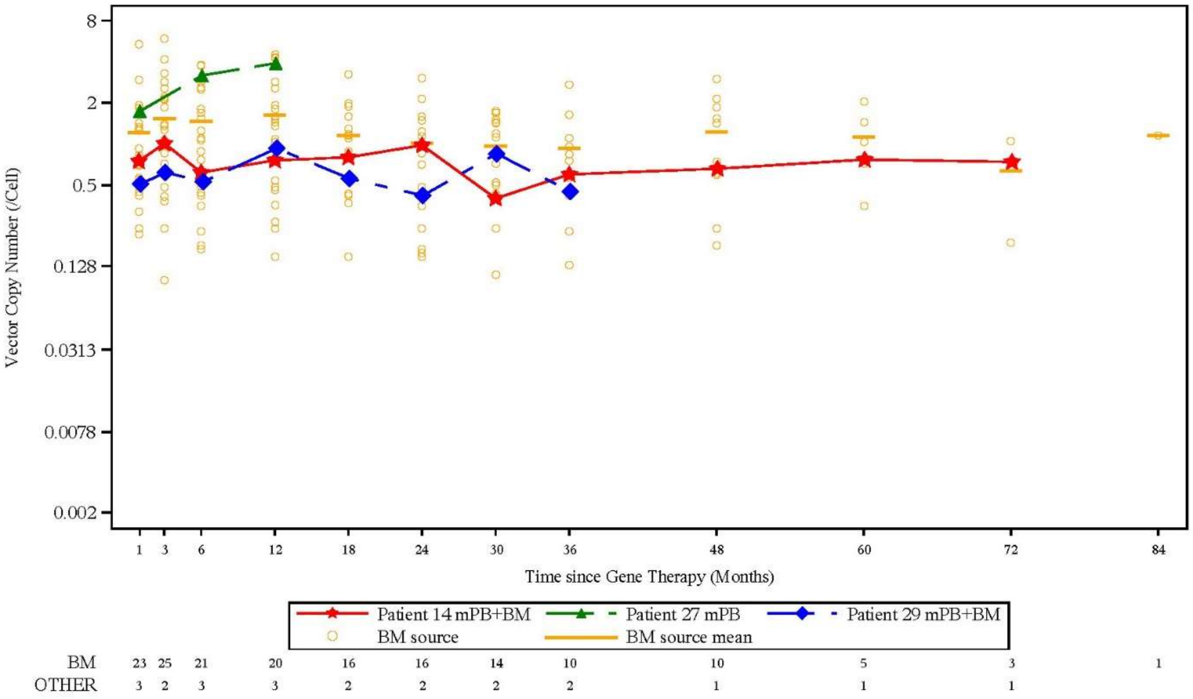

B

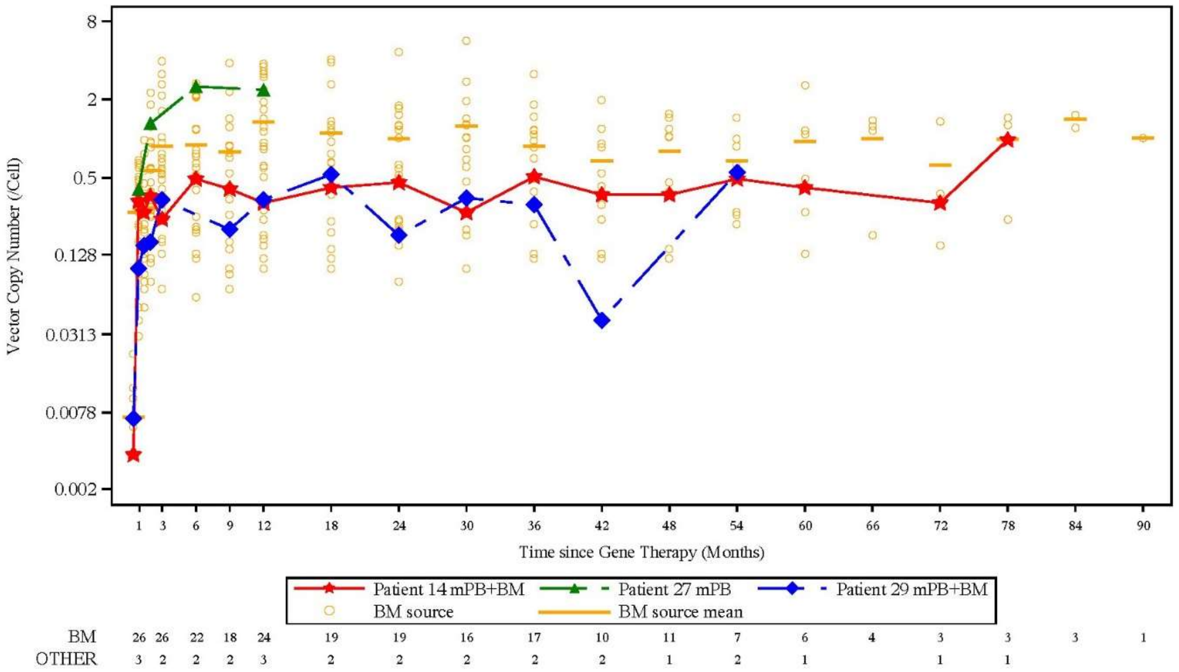

Data from patients 14, 27, and 29 is overlaid on the range of data from patients who received BM-derived CD34+ cells as the sole cell source to manufacture the drug product. Panel B: Due to space limitations, x axis tick marks and patients' frequencies at Day 14, Day 42 and Day 60 have been suppressed. BM=bone marrow; mPB=mobilised peripheral blood.

Figure S6. ARSA activity profiles in myeloid subpopulations of total peripheral blood mononuclear cells. Panel A shows CD14^{+} and Panel B shows CD15^{+}

A

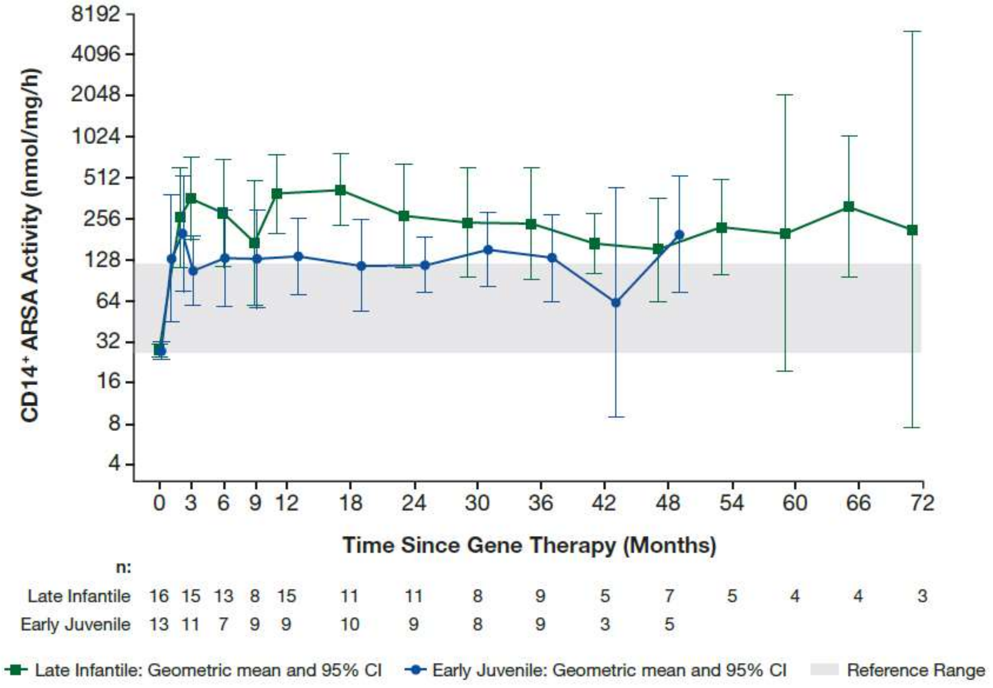

B

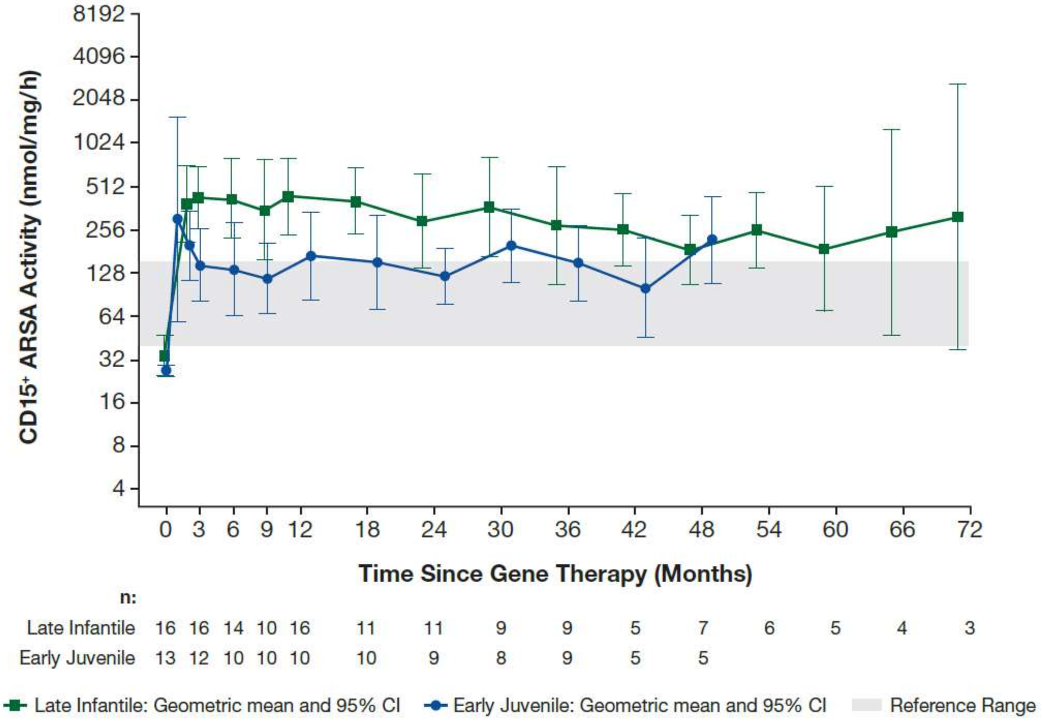

Values <LLOQ were imputed at LLOQ where LLOQ was 25·79 nmol/mg/h. Geometric means and 95% CI are presented at timepoints where there were at least 3 subjects with non-missing data. Reference range represents data from adult reference donors. CI=confidence interval. LLOQ=lower limit of quantitation.

Figure S7. Percentage of lentiviral vector-transduced cells in BM by conditioning regimen (sub-myeloablative or myeloabative)

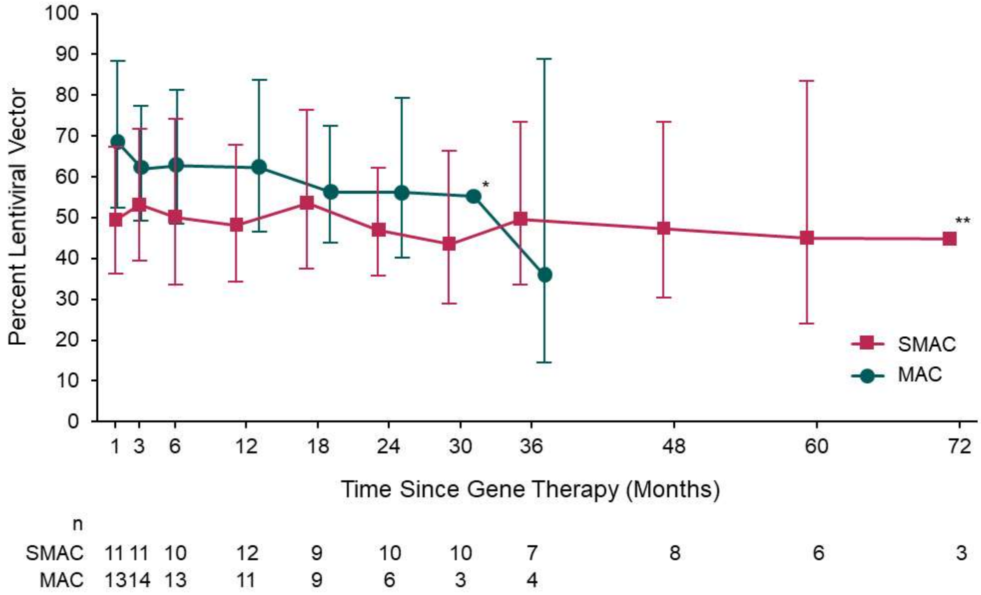

*For MAC at 30 months, 95% CI 21·07–146·79 **For SMAC at 72 months, 95% CI 6·69–303·48. Data points are geometric means with associated 95% CIs and are presented at timepoints where there were at least three subjects with non-missing data. MAC=myeloablative conditioning; SMAC=sub-myeloablative conditioning; CI=confidence interval.

Figure S8. Vector copy number in bone marrow-derived CD34+ cells (Panel A) and peripheral blood mononuclear cells (Panel B) by conditioning regimen

A

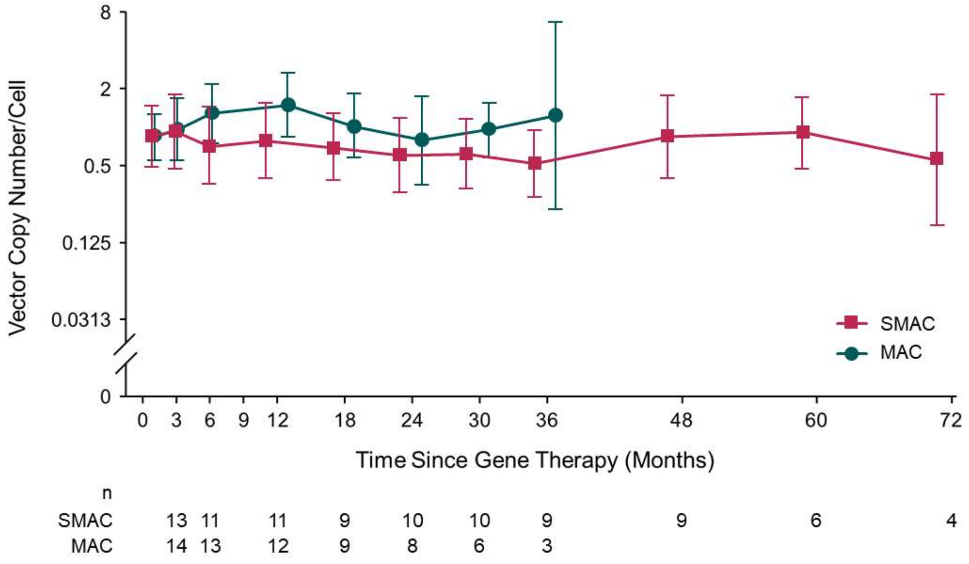

B

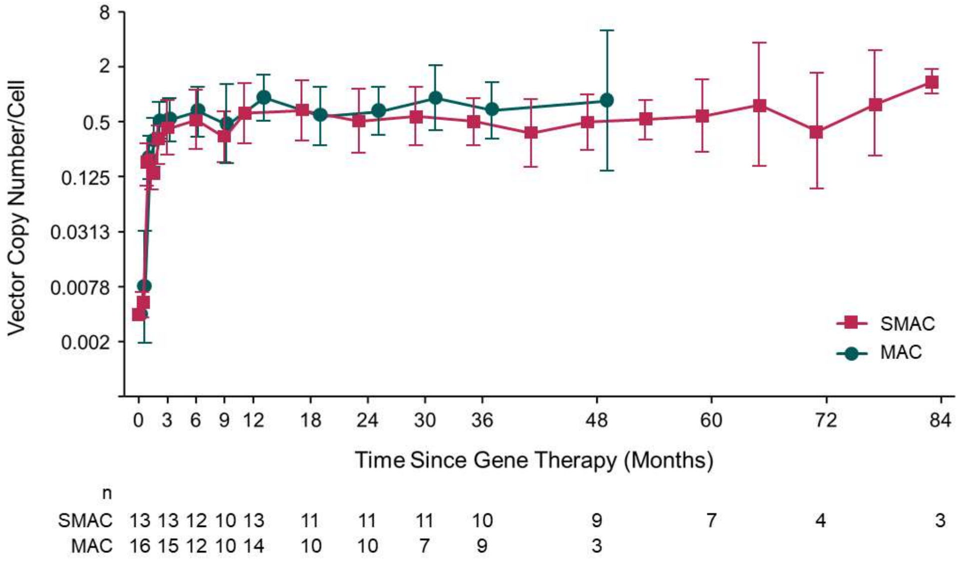

Limit of quantification (LOQ) is 0·0037 VCN/cell. Values < LOQ are imputed at LOQ. Data points are geometric means with associated 95% confidence intervals and are presented at timepoints where there were at least three subjects with non-missing data. MAC=myeloablative conditioning; SMAC=sub-myeloablative conditioning.

Figure S9. ARSA activity profiles in bone marrow-derived mononuclear cells (Panel A) and peripheral blood mononuclear cells (Panel B) by conditioning regimen

A

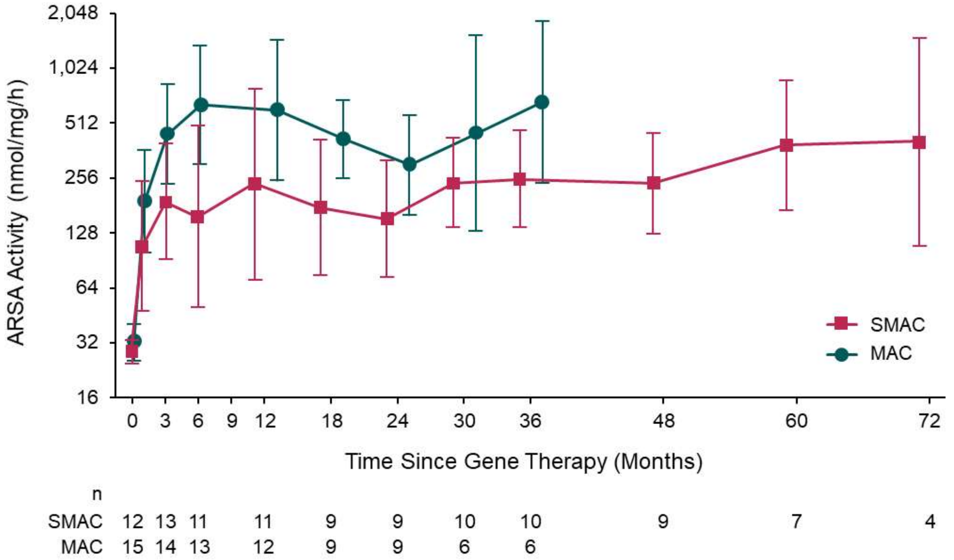

B

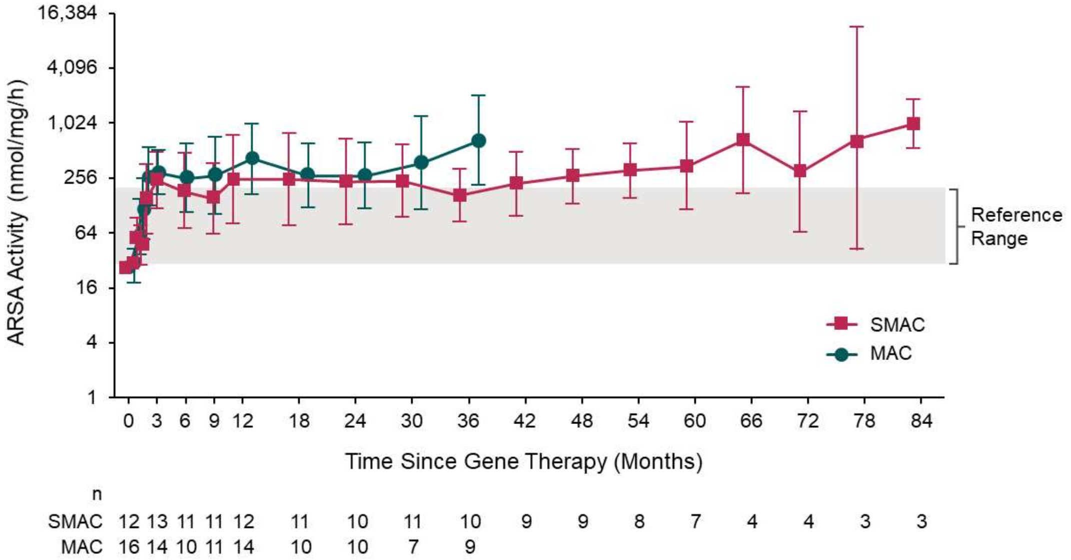

Values <LLOQ were imputed at LLOQ where LLOQ was 25·79 nmol/mg/h. Geometric mean and 95% CI are presented at timepoints where there were at least three subjects with non-missing data. Reference range represents data from adult reference donors. CI=confidence interval. LLOQ=lower limit of quantitation. MAC=myeloablative conditioning; SMAC=sub-myeloablative conditioning

Figure S10. Gross motor function measure total scores after 2 and 3 years in patients with pre-symptomatic (Panel A) and early-symptomatic (Panel B) early-juvenile MLD

A

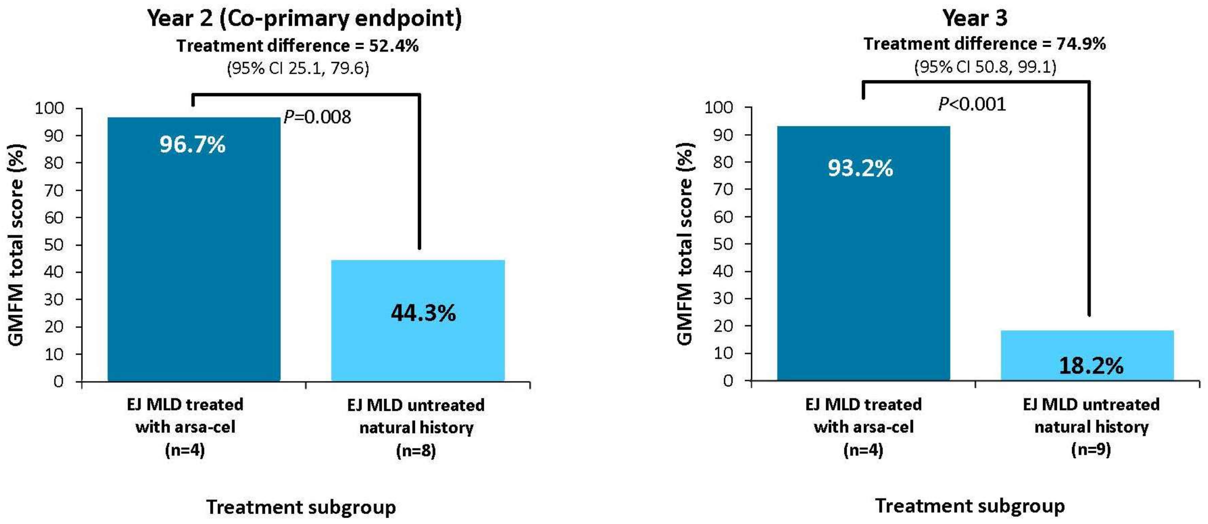

B

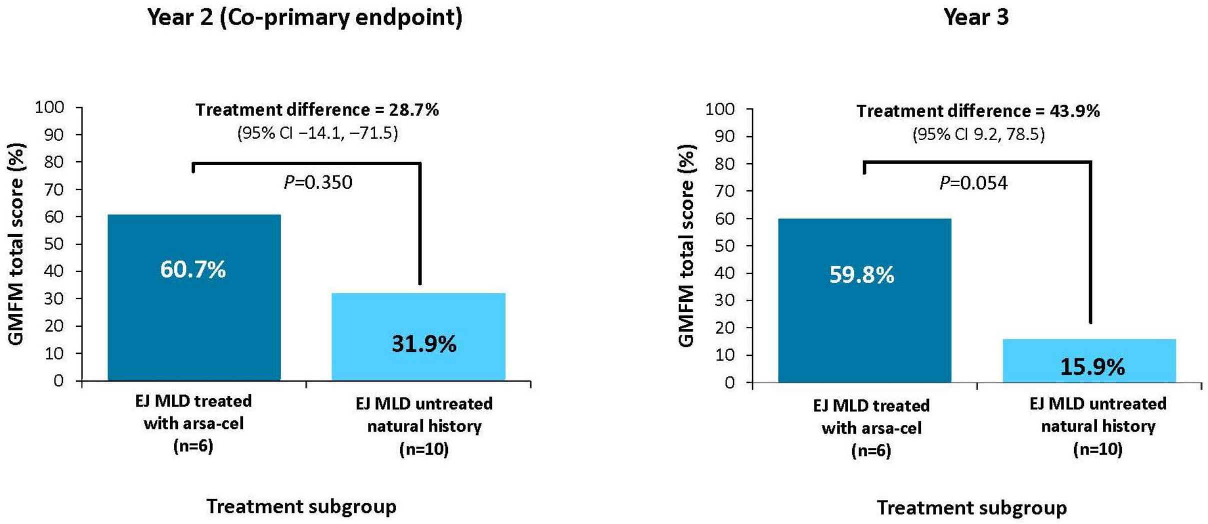

The difference in adjusted least square means are shown (arsa-cel minus natural history). p value for two-sided 5% hypothesis test with null hypothesis of 10% difference. Analysis method for each disease subtype and analysis visit was an analysis of covariance adjusted for treatment and age at GMFM assessment. CI=confidence interval. EJ=early-juvenile. GMFM=gross motor function measure. LI=late-infantile. MLD=metachromatic leukodystrophy.

Figure S11. Panel plots of gross motor function measure score profiles in arsa-cel treated patients with comparison to natural history data, including matched siblings (where applicable). Panel A shows late-infantile, Panel B shows early-juvenile

A

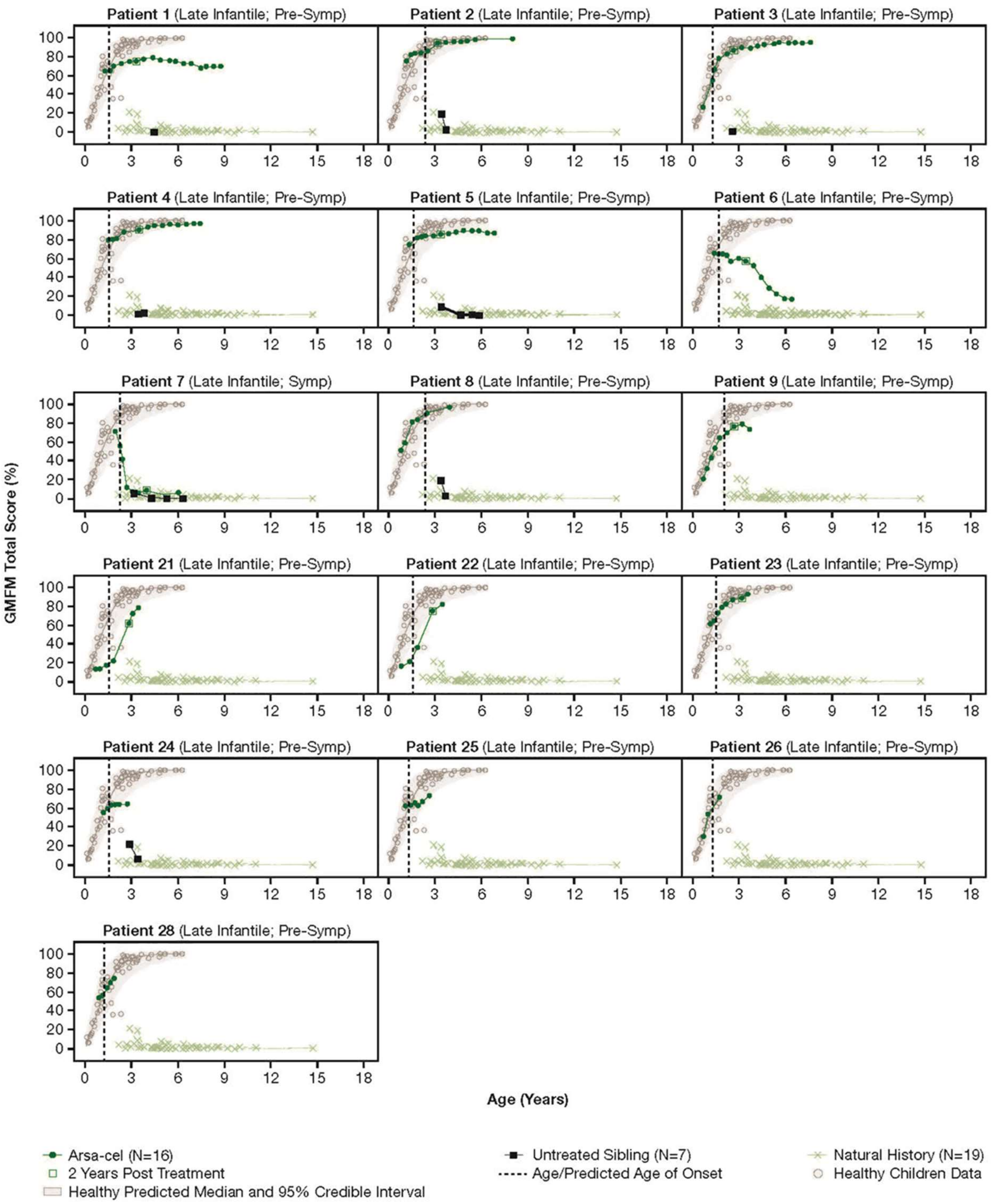

B

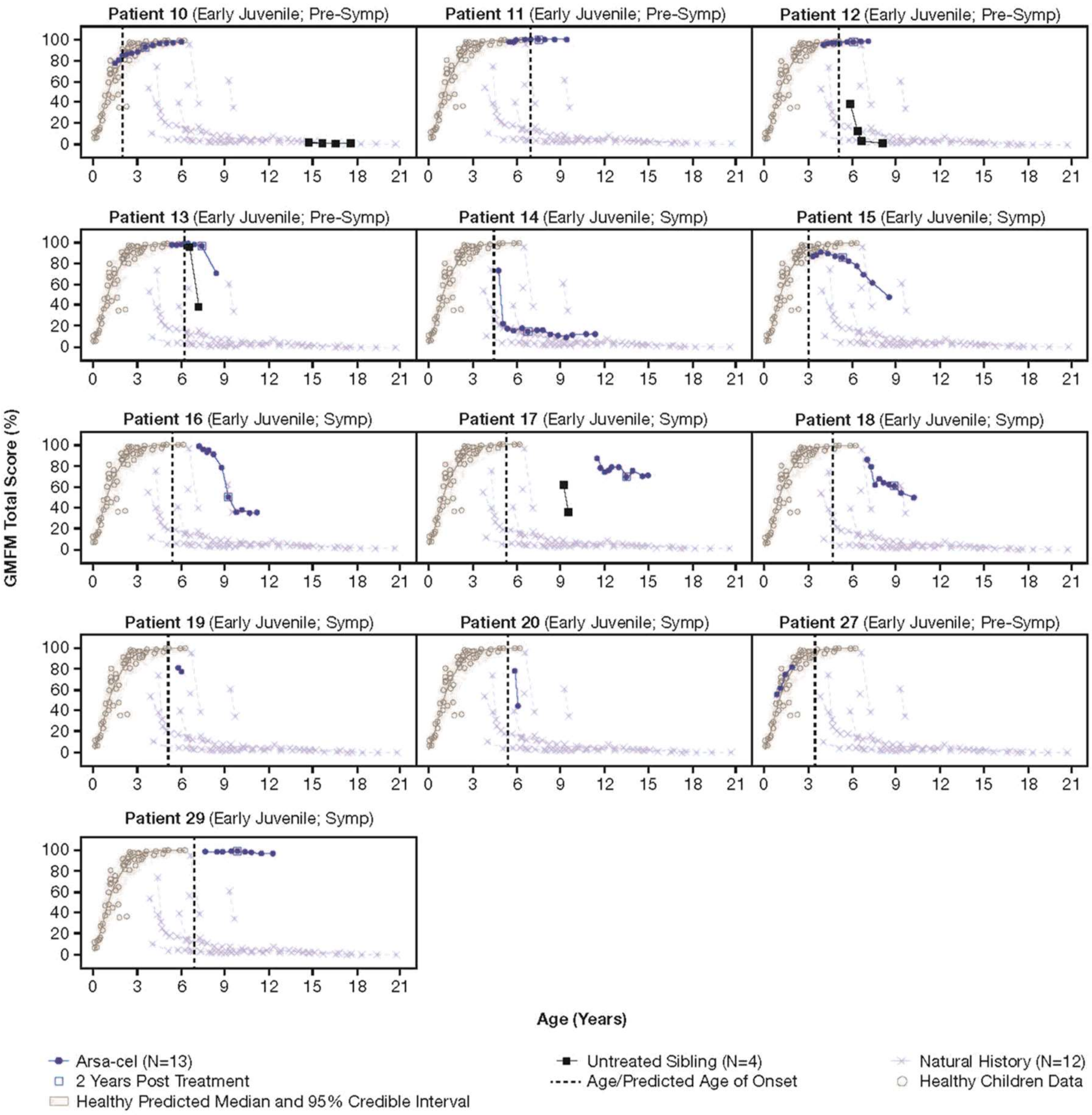

Untreated sibling data are a subset of natural history data, as described earlier in the Supplementary Appendix. Healthy children data are from Dr Palisano and colleagues, who provided access to the anonymous age and GMFM-88 data on 60 subjects in the "No CP" group as reported in Palisano et al. (1997) "Development and reliability of a system to classify gross motor function in children with celebral palsy".[1] Patient 2 and Patient 8 are siblings, and as such have the same untreated sibling. All late-infantile and some pre-symptomatic early-juvenile subjects were identified after an older sibling was diagnosed; data were not available from siblings who were not enrolled in the TIGET NHx study. GMFM=gross motor function measure. Pre-symp=pre-symptomatic. Symp=early-symptomatic.

Figure S12. Panel plots of gross motor function classification levels by age in arsa-cel treated patients compared with natural history data, including matched siblings (where applicable). Panel A shows late-infantile, and Panel B shows early-juvenile

A

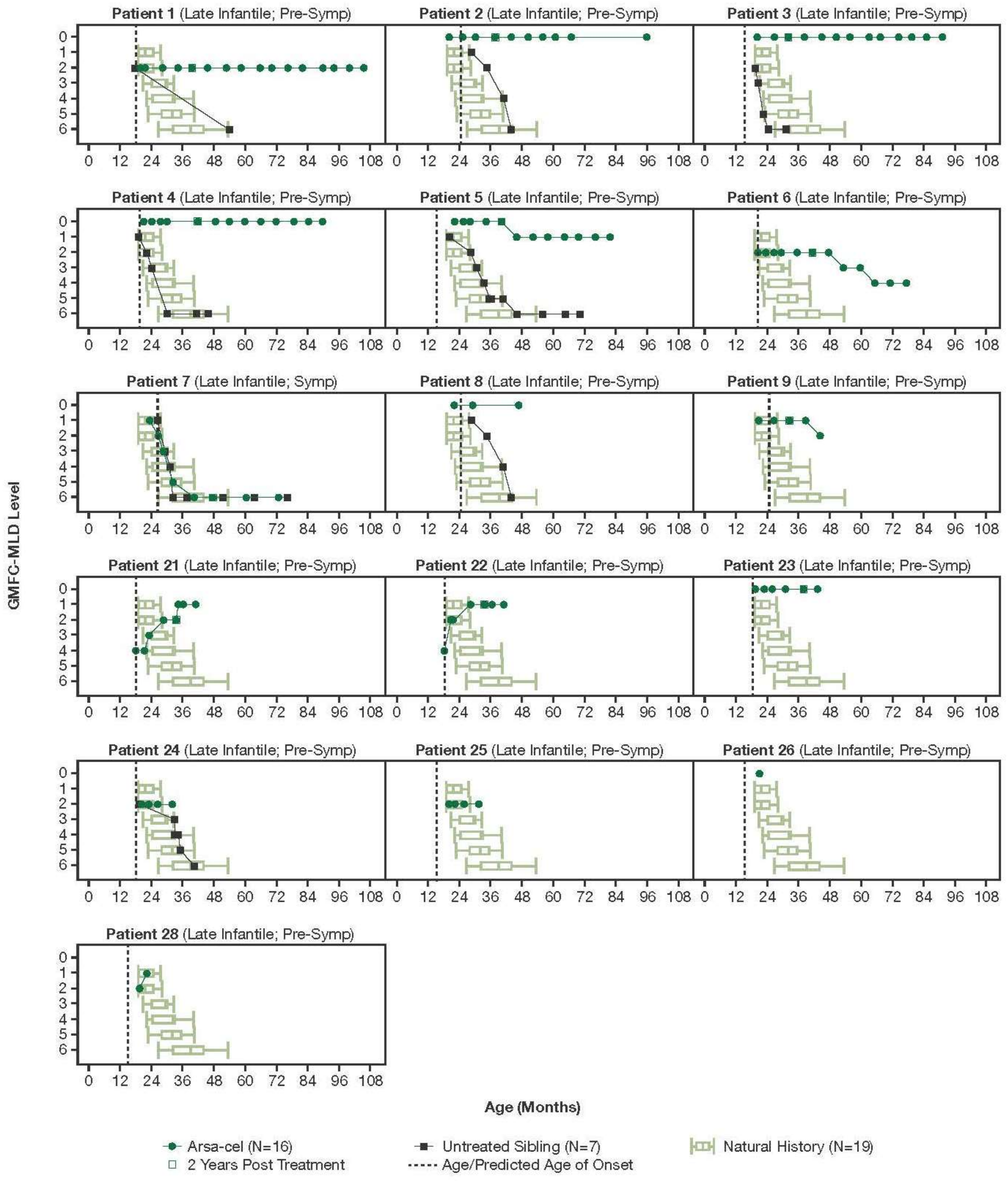

B

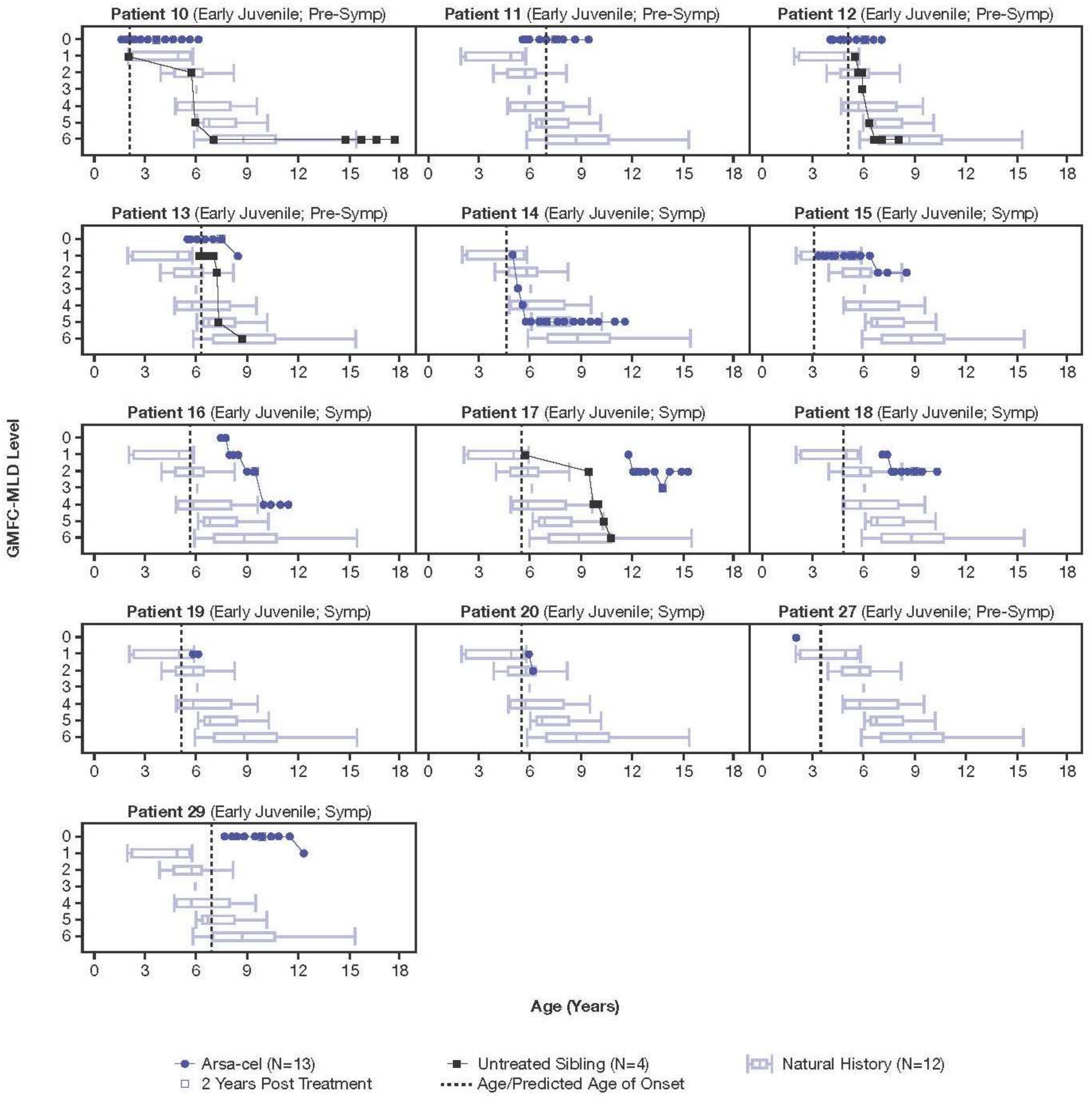

The boxplots display the 10th, 25th, 50th, 75th, and 90th percentiles of the data obtained from the natural history cohort. Untreated Sibling data (when available) is represented by black squares joined by black lines and is a subset of the Natural History data. Treated patient and untreated sibling trajectories have been overlaid on the boxplots. Patient 2 and Patient 8 are siblings, and as such have the same untreated sibling. All late-infantile and some pre-symptomatic early-juvenile subjects were identified after an older sibling was diagnosed; comparison data were not available for siblings who were not enrolled in the TIGET NHx study. Patients 26 and 27 have only one GMFC-MLD level measurement because they have evaluated only once after 18 months of age. The age from which the scale is applicable does not correspond to the baseline evaluation. MLD=metachromatic leukodystrophy. GMFC-MLD=Gross Motor Function Classification-MLD. Pre-symp=pre-symptomatic. Symp=early-symptomatic.

Figure S13. Treatment difference brain MRI scores at 2 and 3 years. Panel A shows late-infantile, and Panel B shows early-juvenile patients

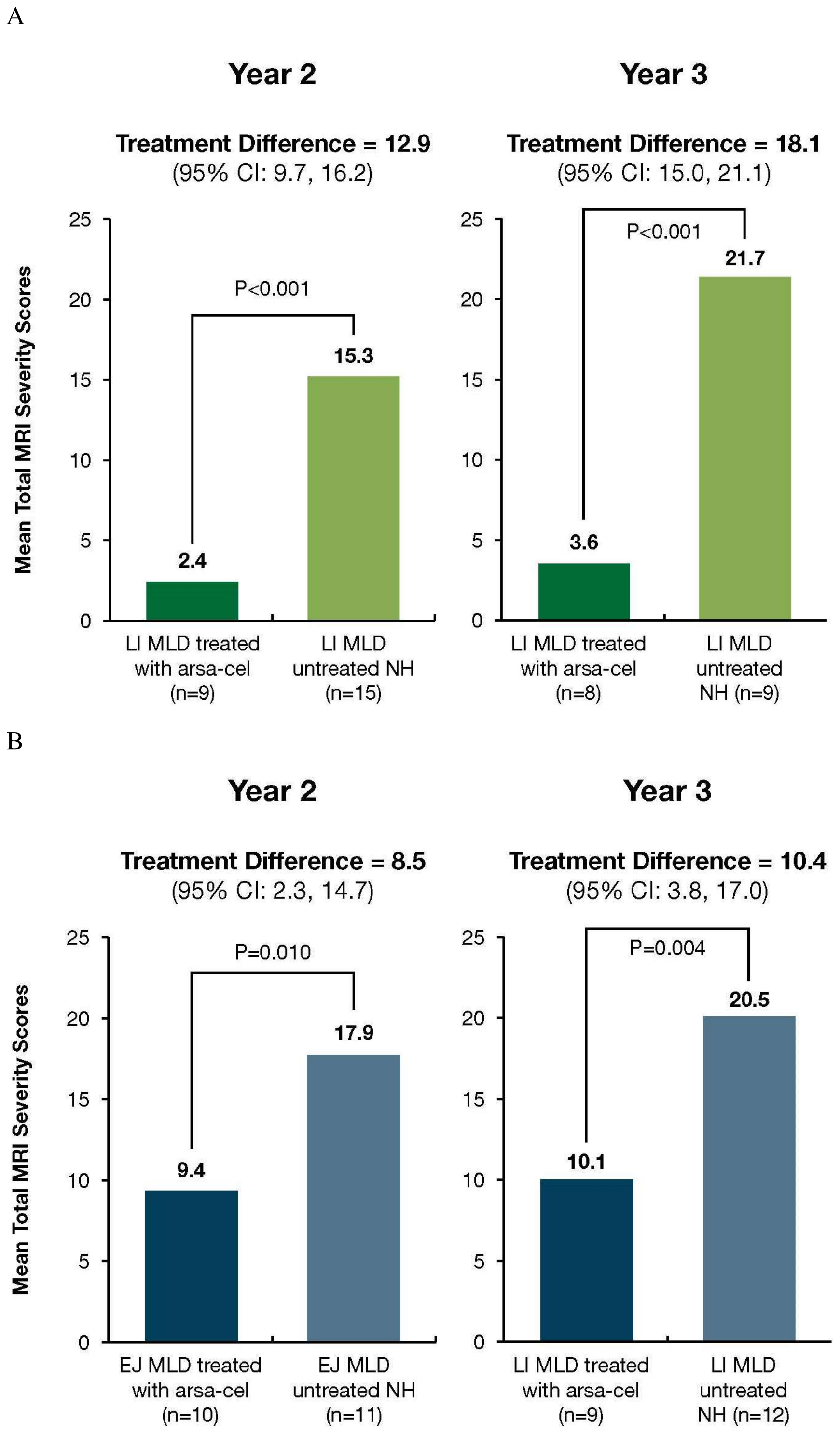

The differences in the adjusted least-square mean magnetic resonance imaging (MRI) total scores between the treated patients and age-matched untreated patients from the natural history study, based on an analysis of covariance model fitting age at MRI assessment and treatment. A positive difference indicates a better outcome in the arsa-cel arm. The analyses show statistically significant treatment differences at Year 2 and Year 3 post-treatment in both late-infantile and early-juvenile patients. indicating stabilisation in treated patients compared with worsening in demyelination and atrophy in Natural History patients, stereotypical of disease progression. MRI severity scoring system is a modified Loes' score as previously described[2]. The maximum total score is 31·5.

Figure S14. Treatment difference NCV Index at 2 and 3 years for late-infantile patients

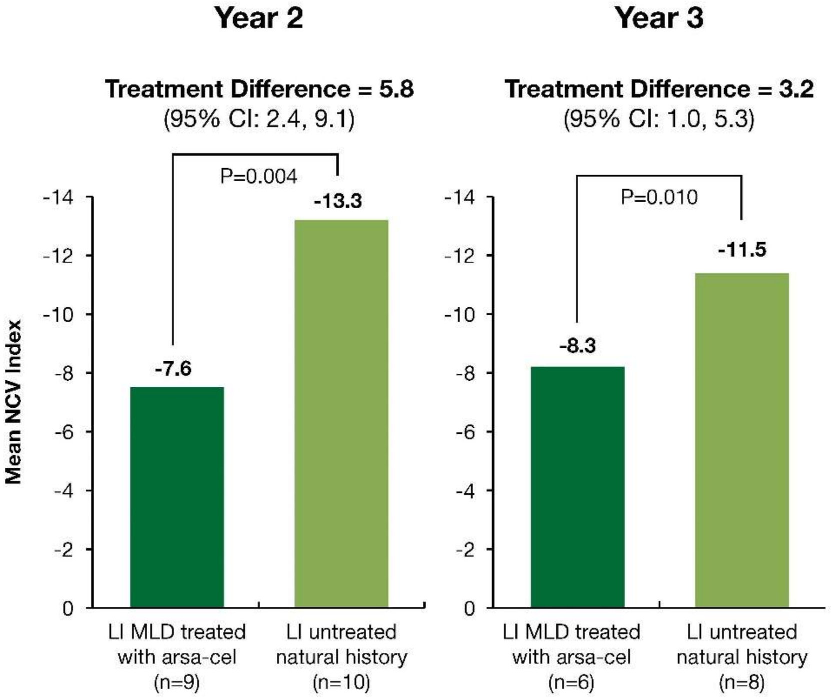

The differences in the adjusted least-square mean NCV Index scores between the treated late-infantile patients and age-matched untreated late-infantile patients from the natural history study, based on an analysis of covariance model fitting age at NCV assessment and treatment. A positive difference indicates a better outcome in the arsa-cel arm. Sensory and motor conduction velocities of four tested nerves were converted to z-scores with a mean of zero and an SD of 1. A Z-score value was calculated by subtracting the mean NCV of an age-matched normal control population from the patient's raw NCV value and then dividing this value by the SD of the normal control population [(patient's NCV − normal controls mean NCV)/normal controls NCV standard deviation]. NCV index was calculated as the average of 4 z-scores from the 4 tested nerves for each patient at each timepoint as previously described.[5,16] A more negative NCV index indicates worsened peripheral neuropathy. LI=late infantile. MLD=metachromatic leukodystrophy. NCV=nerve conduction velocity.

Figure S15. Cognitive age equivalent (performance) for treated patients compared with matched untreated siblings. Panel A shows late-infantile, and Panel B shows early-juvenile patients

A

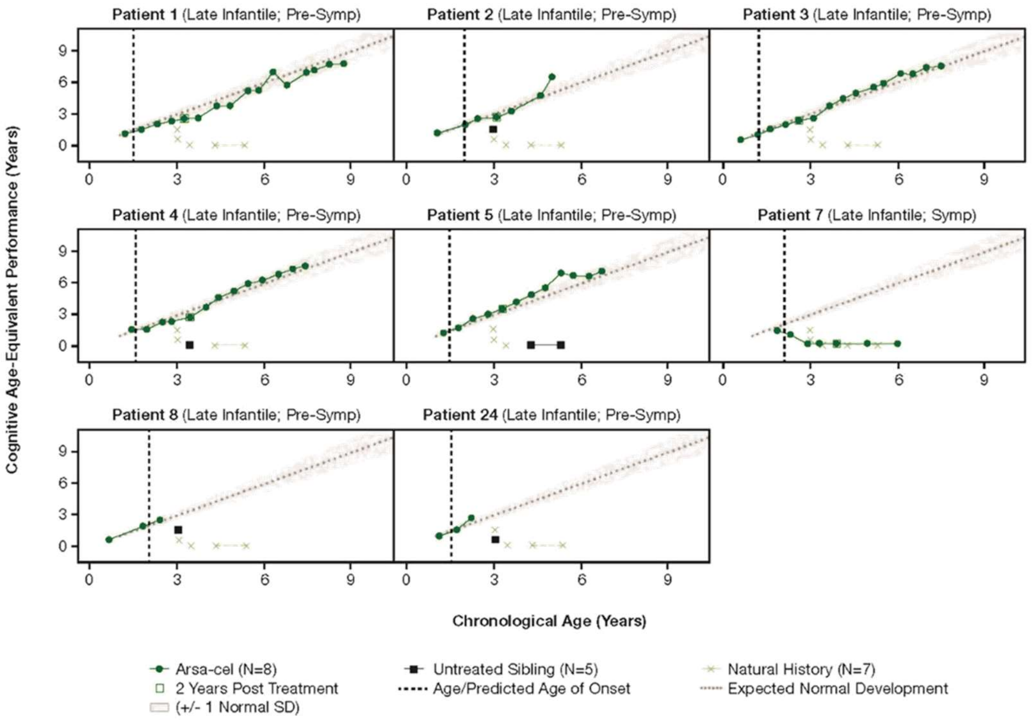

B

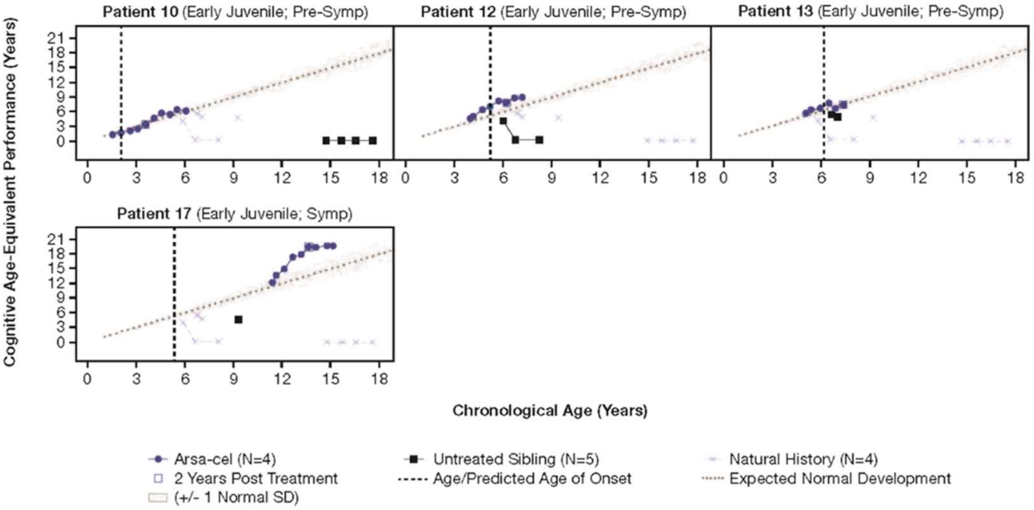

Cognitive age-equivalent performance data were available for 5 late-infantile and 4 early-juvenile treated patients with corresponding untreated siblings. Treated patients showed continued acquisition of cognitive skills as expected for age compared with their corresponding untreated siblings. For the latter, age-equivalents were comparable to those in the overall Natural History population demonstrating cognitive impairment, although lack of longitudinal data precludes the interpretation of the cognitive decline in these patients. Cognitive Age-Equivalent for late-infantile (A) and early-juvenile (B) at each visit has been derived as follows: For WPPSI and WISC: (Development Quotient Performance x Chronological Age)/100 where development quotient is derived by dividing the age-equivalent by the chronological age and then multiplying by 100. For Bayley III: Cognitive Raw Scores have been compared with the tabulated values in the Bayley III manual to calculate Cognitive Age-Equivalent. For Bayley II and in cases where a neuropsychological assessment has been performed but a questionnaire could not be completed due to severe clinical condition, Cognitive Age-Equivalent is based on mental development age as reported on the CRF. SD=standard deviation. Pre-symp=pre-symptomatic. Symp=early-symptomatic.

Figure S16. Kaplan-Meier plot showing age at severe motor impairment or death in treated late-infantile (Panel A) or early-juvenile patients (Panel B) compared with matched untreated siblings

A

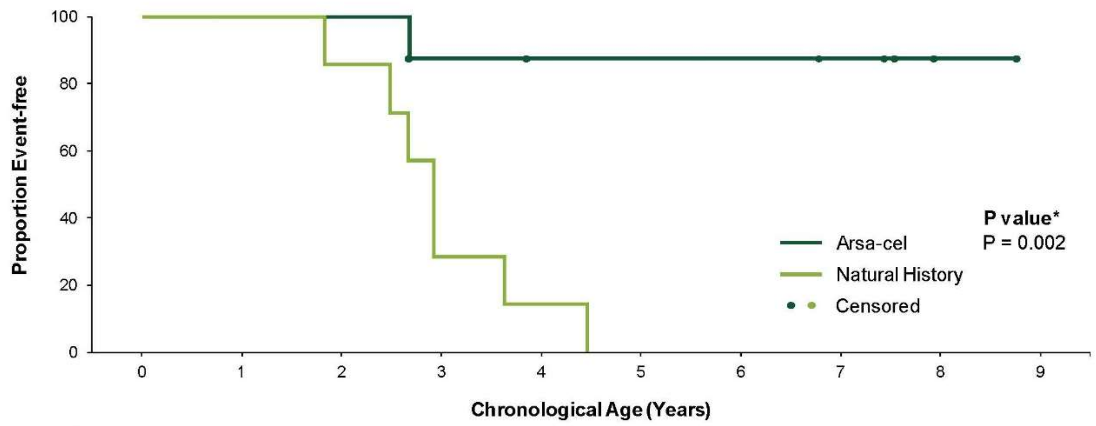

n										
Arsa-cel	8	8	8	6	5	5	5	4	1	0
Natural History	7	7	6	2	1	0	0	0	0	0

*p values calculated using an unstratified log-rank test
Severe motor impairment-free survival (sMFS) is defined as the interval from birth to the earlier of loss of locomotion and sitting without support (GMFC level 5 or higher) or death from any cause; otherwise sMFS is censored at the last GMFC assessment date. Symptomatic status refers to arsa-cel treated subjects at time of treatment. Natural history patients also presented.

B

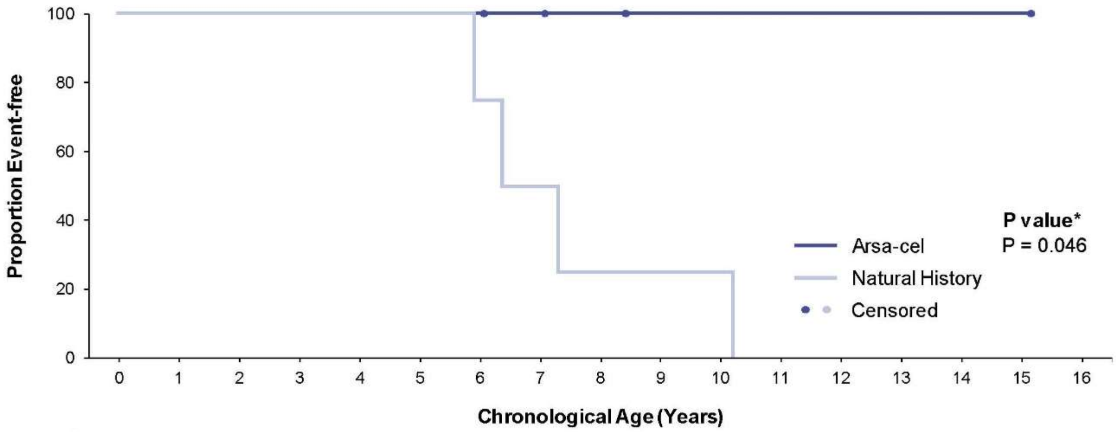

n																	
Arsa-cel	4	4	4	4	4	4	4	3	2	1	1	1	1	1	1	1	0
Natural History	4	4	4	4	4	4	3	2	1	1	1	0	0	0	0	0	0

*p values calculated using an unstratified log-rank test
Severe motor impairment-free survival (sMFS) is defined as the interval from birth to the earlier of loss of locomotion and sitting without support (GMFC level 5 or higher) or death from any cause; otherwise sMFS is censored at the last GMFC assessment date. Symptomatic status refers to arsa-cel treated subjects at time of treatment. Natural history patients also presented.

References

1. Sessa M, Lorioli L, Fumagalli F, et al. Lentiviral haemopoietic stem-cell gene therapy in early-onset metachromatic leukodystrophy: an ad-hoc analysis of a non-randomised, open-label, phase 1/2 trial. Lancet 2016; 388(10043): 476–87.
2. Palisano R, Rosenbaum P, Walter S, Russell D, Wood E, Galuppi B. Development and reliability of a system to classify gross motor function in children with cerebral palsy. Dev Med Child Neurol 1997; 39(4): 214-23.
3. Russell DJ, Rosenbaum PL, Cadman DT, Gowland C, Hardy S, Jarvis S. The gross motor function measure: A means to evaluate the effects of physical therapy. Dev Med Child Neurol 1989; 31(3): 341–52.
4. Russell D, Palisano R, Walter S, et al. Evaluating motor function in children with Down syndrome: validity of the GMFM. Dev Med Child Neurol 1998; 40(10): 693-701.
5. Ruck-Gibis J, Plotkin H, Hanley J, Wood-Dauphinee S. Reliability of the gross motor function measure for children with osteogenesis imperfecta. Pediatr Phys Ther 2001; 13(1): 10-7.
6. Biffi A, Cesani M, Fumagalli F, et al. Metachromatic leukodystrophy – mutation analysis provides further evidence of genotype–phenotype correlation. Clin Genet 2008; 74(4): 349–57.
7. Dali C, Barton NW, Farah MH, et al. Sulfatide levels correlate with severity of neuropathy in metachromatic leukodystrophy. Ann Clin Transl Neurol 2015; 2(5): 518-33.
8. Kehrer C, Blumenstock G, Raabe C, Krageloh-Mann I. Development and reliability of a classification system for gross motor function in children with metachromatic leucodystrophy. Dev Med Child Neurol 2011; 53(2): 156-60.
9. Kehrer C, Blumenstock G, Gieselmann V, Krägeloh-Mann I, On behalf of the German Leukonet. The natural course of gross motor deterioration in metachromatic leukodystrophy. Dev Med Child Neurol 2011; 53(9): 850–5.
10. Albers CA, Grieve AJ. Test Review: Bayley, N. (2006). Bayley Scales of Infant and Toddler Development– Third Edition. San Antonio, TX: Harcourt Assessment. J Psychoed Assess 2007; 25(2): 180-90.
11. Bayley N. Bayley Scales of Infant Development (2nd ed.). San Antonio, TX: Psychological Corporation; 1993.
12. Black MM, Matula K. La valutazione dello sviluppo infantile. Somministrazione e applicazione delle Scale Bayley: Il Mulino; 2005.
13. Woolger C. Wechsler Intelligence Scale for Children-Third Edition (WISC-III). In: Dorfman WI, Hersen M, eds. Understanding Psychological Assessment Perspectives on Individual Differences. Boston, MA: Springer; 2001: 219-33.
14. Kaufman AS, Flanagan DP, Alfonso VC, Mascolo JT. Test Review: Wechsler Intelligence Scale for Children, Fourth Edition (WISC-IV). J Psychoed Assess 2006; 24(3): 278-95.
15. Shapiro EG, Escolar ML, Delaney KA, Mitchell JJ. Assessments of neurocognitive and behavioral function in the mucopolysaccharidoses. Mol Genet Metab 2017; 122s: 8-16.
16. Marcelli D. Psicopatologia del bambino: Elsevier; 2009.
17. Bartelink IH, Lalmohamed A, van Reij EM, et al. Association of busulfan exposure with survival and toxicity after haemopoietic cell transplantation in children and young adults: a multicentre, retrospective cohort analysis. Lancet Haematol 2016; 3(11): e526-e36.
18. Biffi A, Montini E, Lorioli L, et al. Lentiviral hematopoietic stem cell gene therapy benefits metachromatic leukodystrophy. Science 2013; 341(6148): 1233158.
